# Critical role of the CMGC insert sequence for tyrosine autophosphorylation in the protein kinase DYRK1B

**DOI:** 10.1038/s41598-025-33562-x

**Published:** 2025-12-24

**Authors:** Silvia Detro-Dassen, Katharina Schwandt, Philip Helmich, Stefan Düsterhöft, Walter Becker

**Affiliations:** 1 Institute of Pharmacology and Toxicology, RWTH Aachen University, 52074 Aachen, Germany; 2 Institute of Molecular Pharmacology, RWTH Aachen University, 52074 Aachen, Germany

**Keywords:** DYRK1A, DYRK1B, Kinase domain, Autophosphorylation, CMGC insert, AOMS3, Biochemistry, Cell biology, Molecular biology, Plant sciences

## Abstract

Many CMGC group kinases require phosphorylation of a conserved tyrosine residue in the activation loop to achieve catalytic activity. DYRK family members use a distinctive mechanism involving constitutive *cis*-autophosphorylation of this tyrosine. The structural basis of this process has remained unclear, as it occurs while the kinase is still in an inactive conformation, and the tyrosine does not match the known substrate consensus of DYRKs. Here, we exploited the different autophosphorylation capacities of the paralogs DYRK1A and DYRK1B to define structural determinants of this process. DYRK1A efficiently autophosphorylates even in cell-free systems, whereas DYRK1B does not. Using domain swaps and point mutations, we identify the CMGC insert in the C-terminal lobe and two adjacent proline residues (P332/P333 in DYRK1B) as critical for proper folding and activation. Mutation of either proline impaired DYRK1B autophosphorylation and nuclear localization but had no effect in DYRK1A. Substitution of the DYRK1B CMGC insert with that of DYRK1A rescued the maturation defect, demonstrating functional interplay between the insert and flanking prolines. Furthermore, the pathogenic R349W mutation in DYRK1B, associated with monogenic obesity and type 2 diabetes, also disrupted autophosphorylation. These findings highlight the role of the CMGC insert and adjacent prolines in DYRK kinase maturation and autoactivation.

**Supplementary Information:**

The online version contains supplementary material available at 10.1038/s41598-025-33562-x.

## Introduction

The catalytic activity of eukaryotic protein kinases is tightly regulated by reversible transitions between inactive and active conformations, which can be triggered by a wide range of signaling inputs. Many kinases require the phosphorylation within the activation loop of the catalytic domain for their activation^1–3^. This phosphorylation is typically mediated by upstream kinases that act as part of hierarchical signaling cascades.

Paradoxically, certain kinases within the CMGC branch of the kinome—named after the initials of its main kinase families: CDKs, MAPKs, GSKs, and CLKs—bypass this external regulation by undergoing *cis*-autophosphorylation. In this process, the kinase catalyzes the phosphorylation of its own activation loop, enabling constitutive or autonomous activity^4–6^. This raises a fundamental mechanistic question: how can non-phosphorylated kinase molecules, which are supposedly catalytically inactive, initiate autophosphorylation in the absence of prior activation? Even more perplexing, DYRK (dual-specificity tyrosine-phosphorylation-regulated kinase) family kinases autoactivate by the phosphorylation of a conserved tyrosine residue in the activation loop, although the mature kinases phosphorylate their substrates exclusively on serine or threonine residues^4,5^.

For DYRKs, HIPKs (Homeodomain-Interacting Protein Kinases) and GSK3 (Glykogen Synthase Kinase 3), autophosphorylation occurs as a one-time event during protein folding and stabilizes the active conformation of the kinase domain^4,7–11^. This autoactivation is thought to involve a transient folding intermediate with distinct structural properties, which allows the phosphorylation of the tyrosine within the kinase’s own activation loop^4,6,7^. This “prone-to-autophosphorylation” conformation is facilitated by the higher structural flexibility of the unphosphorylated kinase domain compared to the mature, fully active enzyme^8^. Mature DYRK molecules are stoichiometrically autophosphorylated and exhibit constitutive catalytic activity.

In addition to autophosphorylation of the conserved tyrosine residue in the activation loop (Y321), the maturation of DYRK1A also involves phosphorylation of S97 in its N-terminal region^12,13^. Nevertheless, the autophosphorylation of Y321 is an intrinsic capacity of the DYRK1A catalytic domain and can occur independently of non-catalytic regions or auxiliary factors, as demonstrated in cell-free expression systems^8,14^. In contrast, DYRK1B—a closely related paralog of DYRK1A that shares 85% sequence identity within the catalytic domain—exhibits only minimal phosphorylation at the corresponding activation loop tyrosine (Y273) when expressed by in vitro translation^8,15^. Furthermore, pharmacological inhibition of HSP90 chaperone activity selectively impairs the activating autophosphorylation of DYRK1B, but not DYRK1A, in mammalian cells^15^. This dependence on chaperone function suggests that thermodynamic factors contribute to the differential folding and maturation of these kinases. Notably, the crystal structure of the mature, autophosphorylated DYRK1B catalytic domain is virtually indistinguishable from that of DYRK1A^16^, suggesting that the impaired maturation of DYRK1B in bacterial expression systems likely results from unfavorable thermodynamic properties encountered during the folding process, rather than differences in the final active conformation.

A recent study by Lee et al.^17^ introduced a new dimension to our understanding of DYRK1 maturation, identifying a proline residue, P332 in DYRK1B, as essential for tyrosine autophosphorylation, which is conserved in DYRK1A (P380). According to their findings in U87 glioma cells, hydroxylation of P332 by PHD1 (prolyl hydroxylase domain protein 1, encoded by the *EGLN2* gene) precedes and is required for successful autophosphorylation of Y273 in DYRK1B. P332 is located just N-terminal to the so-called CMGC insert region, a structural feature distinctive to CMGC kinases and the region of greatest sequence divergence between DYRK1A and DYRK1B. Interestingly, the proline-containing motif is conserved not only in class I DYRKs (DYRK1A and DYRK1B) but also across many other CMGC kinases. The authors extended their analysis to other kinases, including class II DYRKs, GSK3β and p38α, to support the hypothesis that the formation of prolyl-hydroxylated intermediates may represent a general mechanism of CMGC kinase maturation.

DYRK1A and DYRK1B play key roles in neurodevelopment and cancer biology and are increasingly recognized as potential drug targets in disorders including Alzheimer’s disease, diabetes mellitus, metabolic syndrome, heart disease, and cancer^18–22^. In the present study, we set out to investigate the mechanism underlying the maturation of DYRK kinases by tyrosine autophosphorylation. Specifically, we focused on the role of the essential proline residue adjacent to the CMGC insert and conducted structure–function analyses to delineate the sequence elements responsible for the differing autoactivation characteristics of DYRK1A and DYRK1B. In addition, we examined the functional consequences of a pathogenic point mutation (R349W) within the CMGC insert of DYRK1B.

## Results

### Mammalian expression systems

To investigate the roles of P332 and the adjacent P333 in DYRK1B maturation, as well as the corresponding residues in DYRK1A (P380 and P381), we overexpressed point mutants of GFP-tagged DYRK1A and DYRK1B in HeLa and HEK293 cells. To assess the potential involvement of prolyl hydroxylation, we used the PHD inhibitor Roxadustat (a.k.a. FG-4592). Activation loop phosphorylation—Y321 in DYRK1A and Y273 in DYRK1B—was detected by immunoblotting and served as a molecular marker of successful kinase folding and maturation.

**Fig. 1:**
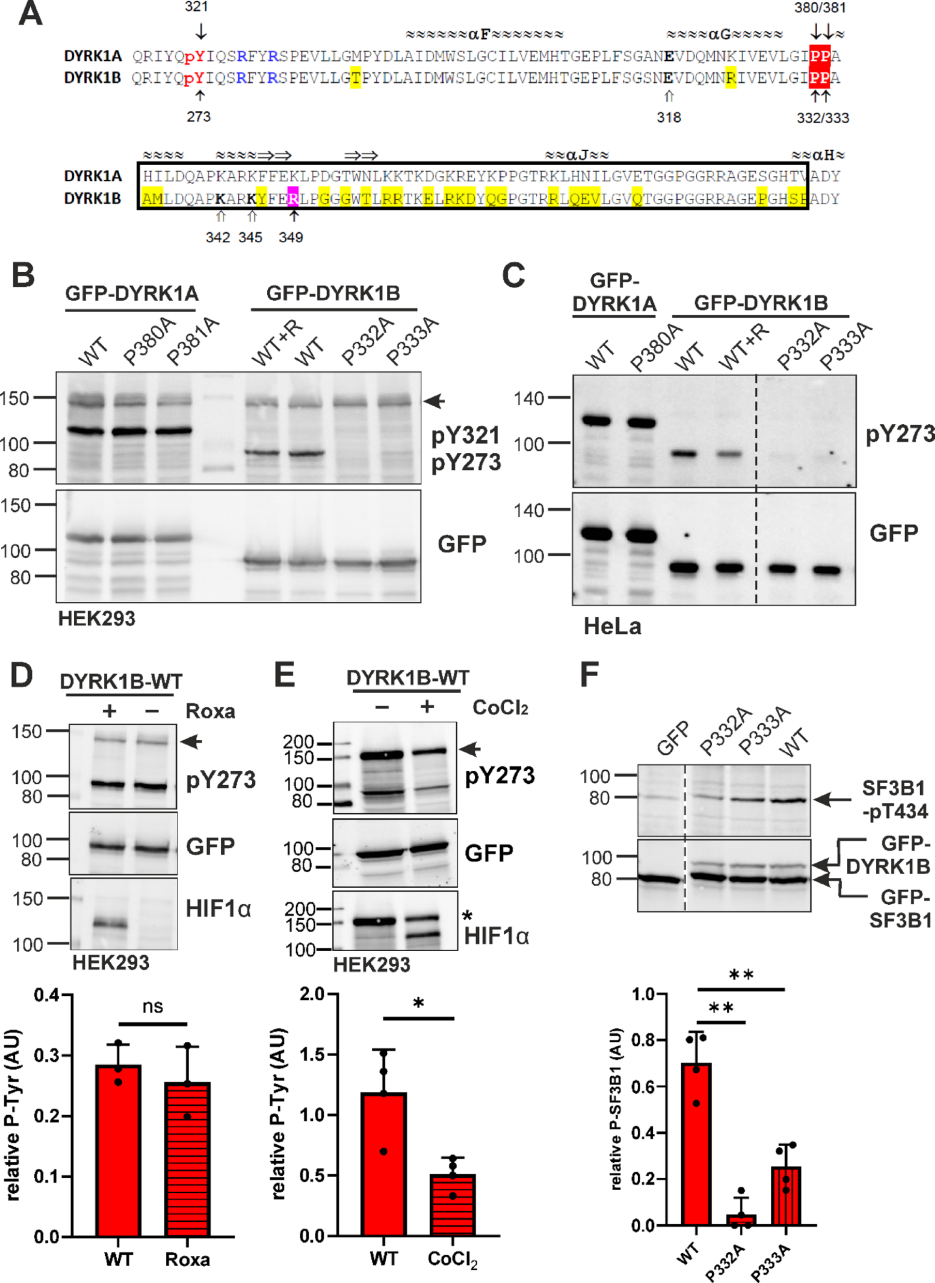
Maturation of DYRK1A and DYRK1B in mammalian cells. (**A**) Sequence alignment of DYRK1A and DYRK1B. The alignment includes the part of the catalytic domain from the autophosphorylation site (pY321/pY273) and the two arginine residues that coordinate the phosphotyrosine (blue) up to the double proline motif (red inverse print) and the CMGC insert sequence (boxed). The CMGC insert is located between helix αG and helix αH of the kinase domain and includes helix αJ and an antiparallel β-sheet (indicated by arrows). The position of a pathogenic DYRK1B variant (R349W) is highlighted (magenta). Open arrows point to K342 and K345 in DYRK1B (see Fig. 6). The region swapped in chimera 3 is boxed (see Fig. 5); it contains 23 of the 46 amino acid differences found between DYRK1A and DYRK1B across the entire catalytic domain (shaded in yellow). (**B**) Tyrosine autophosphorylation of transiently expressed GFP-DYRK1 constructs was assessed by Western blot analysis of total cell lysates. Five hours after transfection, Roxadustat (100 µM) was administered to one well of cells with WT DYRK1B (WT + R). Phosphorylation of the tyrosine in the activation loop (Y321 in DYRK1A, Y273 in DYRK1B) was detected with the help of a pY361(HIPK2) antibody that crossreacts with the activating phosphotyrosine in DYRK1^15,44^. The arrowhead points to the endogenous HIPK2 band. The image shows a representative result of 3 biological replicates. (**C**) GFP-DYRK1 constructs were immunoprecipitated from transiently transfected HeLa cells. The dashed line indicates where irrelevant lanes were deleted from the final image (representative for 3 biological replicates). (**D**, **E**), Transiently transfected HEK293 cells were treated with Roxadustat (100 µM) or CoCl_2_ (300 µM). HIF1α was detected to validate effective PHD inhibition. The column diagrams show the quantitative evaluation of the pY273 signal relative to the GFP signal (means and SD; n = 3). The band marked by the asterisks reappeared from the preceding detection of HIPK2. (**F**) HEK293 cells were transiently transfected to co-express GFP-SF3B1 and GFP-DYRK1B constructs. Phosphorylation of SF3B1 on T434 was detected by immunoblot analysis with a phosphospecific antibody. Relative catalytic activity of the overexpressed DYRK1B constructs was determined by normalizing the pT434 signal to total GFP-SF3B1. Background pT434 phosphorylation by endogenous DYRK1 was subtracted.

Unexpectedly, mutation of either proline residue (P332 or P333) abolished tyrosine autophosphorylation in DYRK1B, while the phosphotyrosine signal of DYRK1A remained unaffected (Fig. 1B, C). We detected no apparent reduction in phosphotyrosine levels following treatment with the PHD inhibitor Roxadustat.

To confirm and quantify the latter observation, we performed additional experiments using Roxadustat. Robust induction of hypoxia-inducible factor 1 (HIF1) confirms effective inhibition of prolyl hydroxylases, yet Roxadustat had no significant effect on the phosphotyrosine content of GFP-DYRK1B in HEK293 cells (Fig. 1D). In contrast, treatment with the hypoxia-mimetic cobalt chloride—previously used by Lee et al.^17^ in U87 glioma cells—produced a marked reduction in DYRK1B phosphotyrosine levels (Fig. 1E). Interestingly, the tyrosine autophosphorylation of endogenous HIPK2 was reduced to a similar extent. We initially hypothesized that CoCl_2_ might directly inhibit DYRK1B autophosphorylation; however, in vitro-assays revealed no effect of CoCl_2_ on substrate phosphorylation nor on tyrosine autophosphorylation (see supplementary Fig. S1).

Next we asked whether the proline mutations affect the catalytic activity of DYRK1B towards a substrate protein. Phosphorylation of splicing factor 3B1 (SF3B1) on T434 is a marker of cellular DYRK1 activity^23^ and has been previously used to characterize the impact of *DYRK1B* mutations^24^. Following correction for background phosphorylation by endogenous DYRK1A and DYRK1B, our analysis revealed that the P332A mutation left only minimal residual activity, whereas the P333A mutant retained approximately 20% residual activity compared to wild-type DYRK1B (Fig. 1F).

**Fig. 2:**
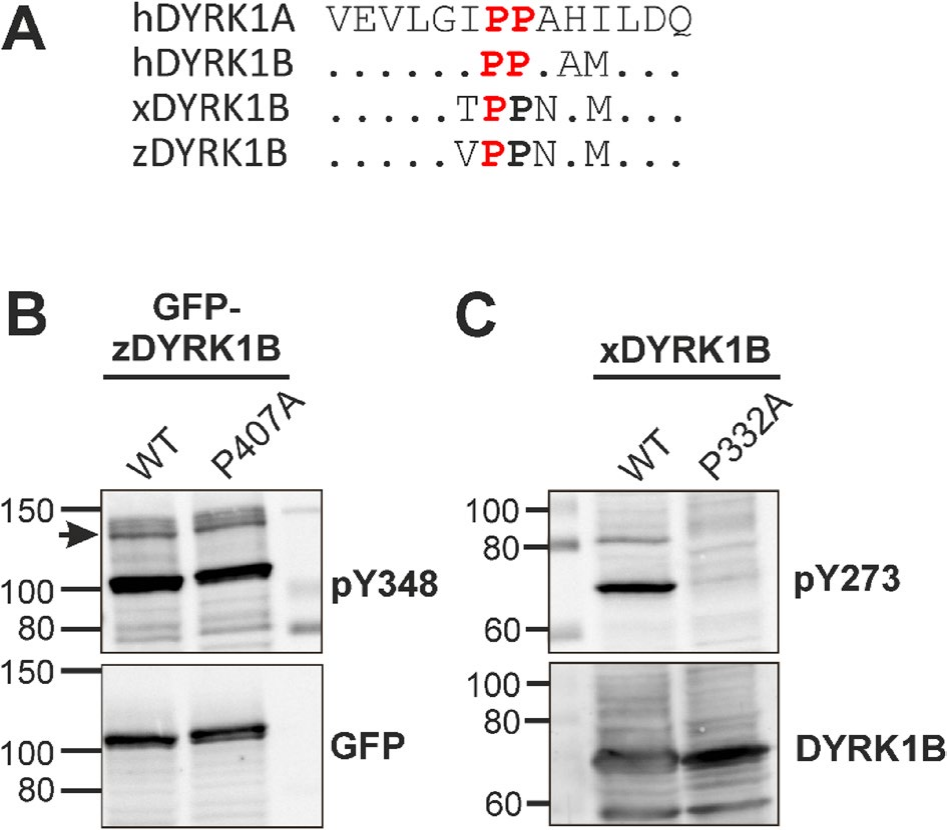
Effect of the proline mutation in non-mammalian DYRK1B orthologs. (**A**) Sequence conservation of the proline motif. The arrow points to P380 in human DYRK1A (sequence ID NP_001387.2), which corresponds to P332 in human DYRK1B (NP_004705.1), P332 in *Xenopus laevis* DYRK1B (NP_001080262.1), and P407 in zebrafish DYRK1B (XP_005158272.1). The prolines that were functionally characterized by mutagenesis are shown in red. (**B**,** C**) DYRK1B constructs from Zebrafish (zDYRK1B) (**B**) and *Xenopus laevis* (xDYRK1B) (**C**) were expressed in HEK293 cells. The image shows immunoblot results from total cellular lysates and is representative of two experiments.

Given the evolutionary conservation the proline motif—but the divergent functional effects of P332A in DYRK1B and P380A in DYRK1A—we investigated the impact of this substitution in DYRK1B orthologs from other vertebrate species (Fig. 2). Interestingly, DYRK1B from zebrafish (*Danio rerio*) retained robust tyrosine autophosphorylation despite the P407A mutation, whereas the corresponding P332A mutation in *Xenopus* DYRK1B nearly eliminated autophosphorylation.

In aggregate, these findings indicate that P332 plays a critical role in the maturation of human and frog DYRK1B, but is not universally required for tyrosine autophosphorylation among class I DYRKs.

### Subcellular localization

**Fig. 3:**
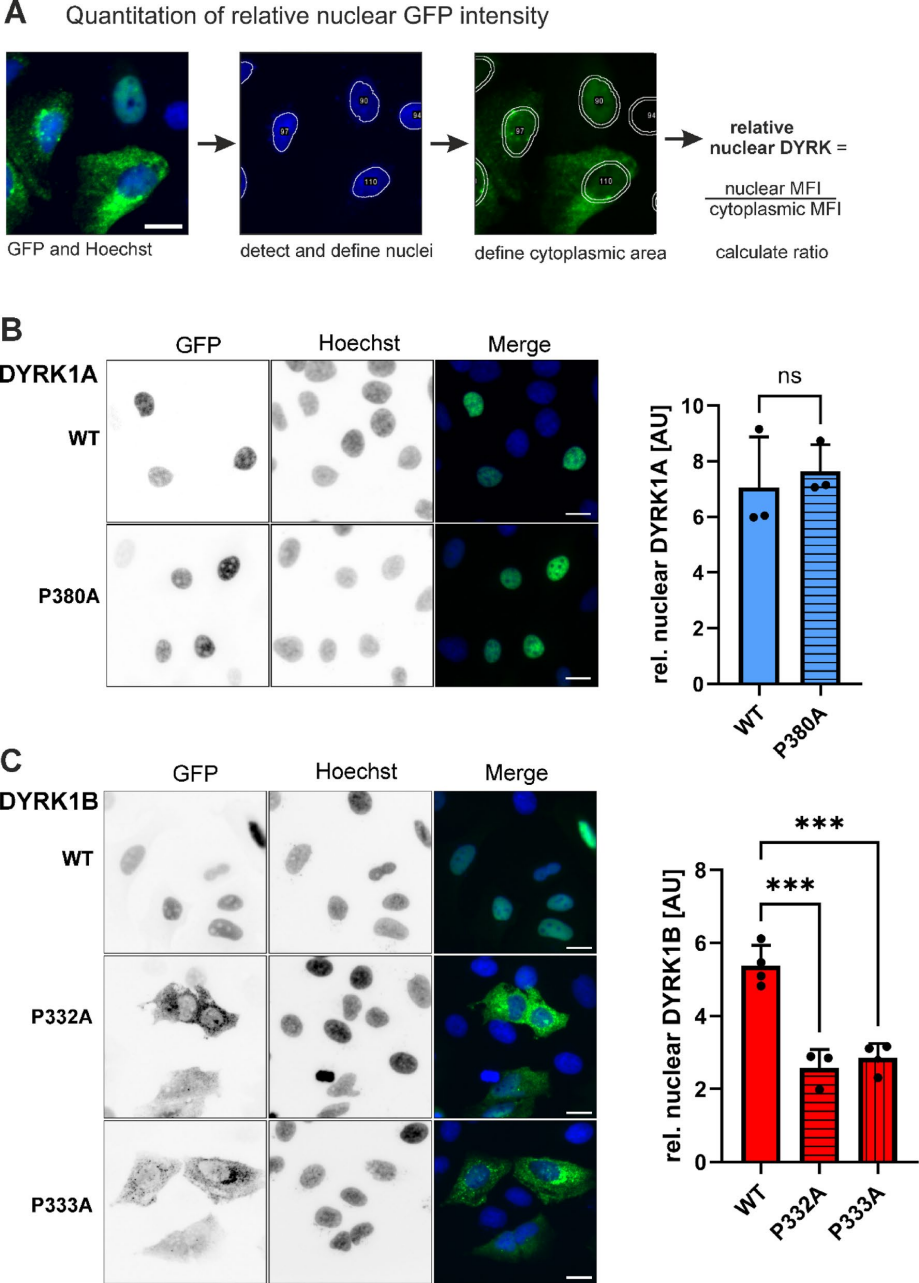
Subcellular localization of GFP-DYRK1 proline mutants. Fluorescence microscopic analysis of U2OS cells transiently expressing wild type or mutant GFP-DYRK1 constructs. (**A**) Quantification of DYRK1 subcellular distribution using an automated nuclear–cytoplasmic (N/C) ratio analysis script (ImageJ/FIJI macro). Nuclei (N) were identified by Hoechst staining. As a proxy for cytoplasmic localization (**C**), a ring was defined by expanding the boundary of the nucleus (N). The N/C ratio of the mean fluorescence intensities (MFI) was used as a measure for the relative proportion of the GFP fusion protein in the nucleus. (**B**, **C**) Representative images and quantitative evaluation of GFP-DYRK1A (**C**) and GFP-DYRK1B (**B**) constructs. At least 60 transfected cells were evaluated per data point in three independent experiments (scale bars, 20 μm).

Mutant DYRK1 molecules that fail to mature into their active conformation form extranuclear aggregates, as detectable by fluorescence microscopy^17,25^. To investigate whether the proline mutations affect nuclear localization of DYRK1A and DYRK1B, we analyzed GFP-tagged DYRK1 constructs by fluorescence microscopy in U2OS cells (Fig. 3A).

Consistent with the findings of Lee et al.^17^, we observed that GFP-DYRK1B-P332A accumulated in the cytoplasm and showed reduced nuclear localization (Fig. 3C). However, contrary to their observations in U87 glioma cells—where the P333A mutant resembled wild-type DYRK1B—we found that P333A also failed to translocate to the nucleus and was indistinguishable from the P332A mutant. In contrast, DYRK1A-P380A retained normal nuclear localization similar to the wild-type protein (Fig. 3B), with no signs of aggregate formation. These findings indicate that impaired nuclear localization of DYRK1B proline mutants is linked to their reduced phosphotyrosine content and defective maturation, leading to cytoplasmic accumulation.

### Bacterial expression systems

**Fig. 4:**
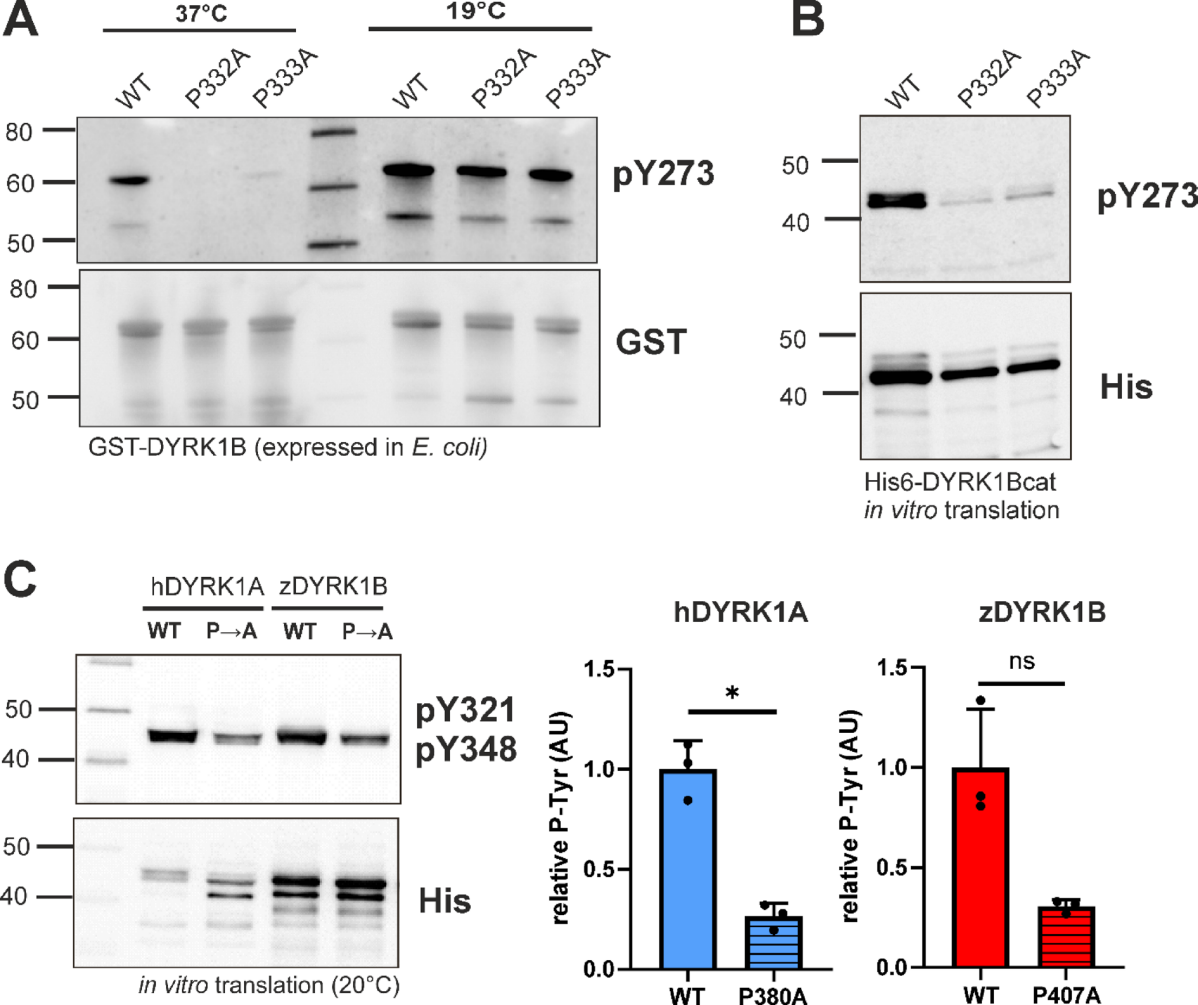
Maturation of DYRK1A and DYRK1B in bacterial expression systems. (**A**) GST-DYRK1B-ΔC constructs were expressed in E. coli at 37 °C for 4 h or at 19 °C for 24 h. The GST fusion proteins were partially purified by affinity adsorption to glutathione Sepharose before Western blot analysis. This result was reproduced with independent preparations of GST-DYRK1A and GST-DYRK1B (see supplementary Fig. S2). (**B**, **C**), His_6_-tagged DYRKcat constructs were expressed by coupled in vitro transcription and translation for 4 h at 20 °C. The image in panel (**B**) is representative of 4 experiments. The column diagrams in panel (**C**) illustrate the quantitative evaluation of n = 3 experiments (ratio of pTyr to His_6_ signal, means and SD). hDYR1A, human DYRK1A; zDYRK1B, zebrafish DYRK1B.

To rule out potential confounding factors such as cell type–specific expression of chaperones or prolyl hydroxylases, we investigated the role of P332 and P333 in DYRK1B maturation using *E. coli*-based expression systems. While some bacteria can hydroxylate free proline, native posttranslational proline hydroxylation is absent in *E. coli*^26^. Building on our previous observation that tyrosine autophosphorylation of GST-DYRK1B fusion proteins is highly temperature-dependent^15^, we compared the effects of proline mutations at 37 °C and 19 °C (Fig. 4A). At 37 °C, mutation of either proline effectively abolished tyrosine autophosphorylation, with DYRK1B-P333A showing a minimal residual signal. In contrast, both mutants exhibited efficient maturation at 19 °C. These findings suggest that the critical function of P332 is primarily related to the thermodynamic conditions during protein folding, rather than to hydroxylation by PHD1.

We next examined the effects of the proline mutations using an *E. coli*-based cell-free expression system. To focus on the autonomous folding behavior of the catalytic domain, we employed His₆-tagged DYRK1B constructs lacking the N- and C-terminal regions (His₆-DYRK1Bcat). Expression was only performed at 20 °C, as DYRK1Bcat was insufficiently synthesized at higher temperatures in this system. As shown in Fig. 4B, tyrosine autophosphorylation was markedly reduced in both proline mutants compared to the wild-type protein, indicating that these mutations directly impair the maturation process at the molecular level. Unexpectedly, mutation of the first proline also compromised tyrosine autophosphorylation in human DYRK1A and zebrafish DYRK1B (Fig. 4C), although these mutants showed no defect when expressed in mammalian cells (Fig. 1). These findings suggest that this conserved proline contributes broadly to the maturation of class I DYRKs, but that the consequences of its mutation become apparent only under the difficult thermodynamic conditions of in vitro expression.

### Structure–function analysis of DYRK1A-DYRK1B chimera

**Fig. 5:**
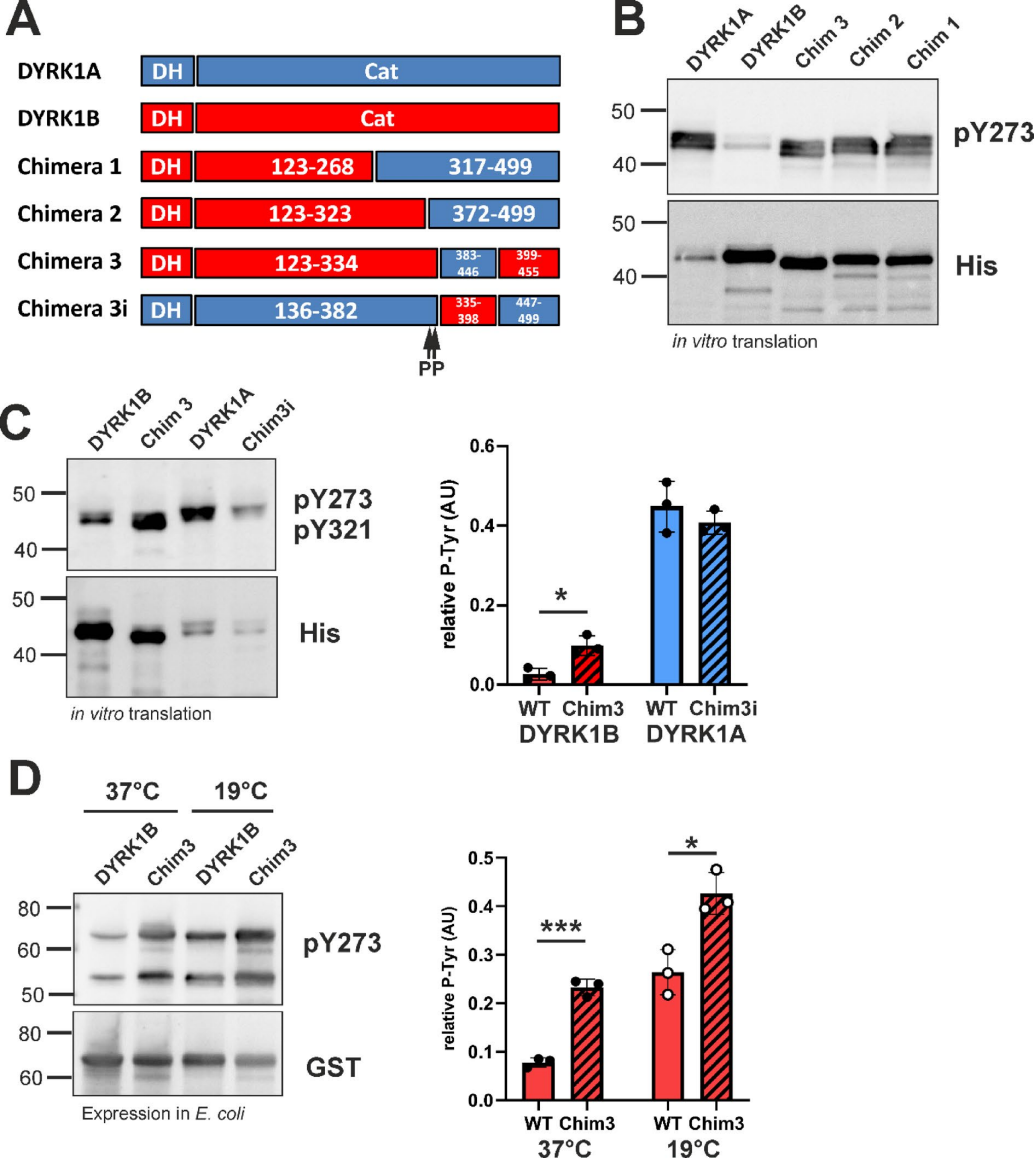
Role of the CMGC insert in the maturation of DYRK1A and DYRK1B. (**A**) Schematic representation of the chimeric constructs of DYRK1A und DYRK1B. The DYRK homology box (DH) is not part of the conserved kinase domain (cat) but is required for efficient maturation.. The position of the double proline motif is marked below chimeric construct Chim 3i. See Fig. 1A for the exact sequences of the regions swapped between DYRK1A and DYRK1B in chimeras 3 and 3i. (**B**, **C**) His_6_-tagged DYRKcat constructs were expressed by coupled in vitro transcription and translation for 4 h at 20 °C. (**D**) GST-DYRK1B-ΔC fusion proteins were expressed in *E. coli* at 37 °C for 4 h or at 19 °C for 23 h. The blots in panel C and D illustrate representative experiments, and the column diagrams show the means and SD of n = 3 experiments.

In our previous work, we used chimeric constructs of DYRK1A and DYRK1B to demonstrate that the divergent ability of these kinases to fold autonomously in the absence of chaperones is primarily determined by sequence differences within the C-lobe of the catalytic domain. Building upon this approach, we generated additional chimeras to further narrow down the critical sequence responsible for this property (Fig. 5A). Cell-free expression of these constructs revealed that substituting a 64-amino-acid segment (residues 383–446 in DYRK1A, corresponding to 335–398 in DYRK1B) markedly enhanced autophosphorylation of the DYRK1B-based chimera compared to wild-type DYRK1B (chimera 3, Fig. 5B). This segment largely encompasses the CMGC-specific insert region, the most divergent region of the catalytic domain between DYRK1A and DYRK1B (see Fig. 1A).

To further assess the functional relevance of this region, we also constructed the reciprocal chimera (Chim 3i), in which the CMGC insert of DYRK1A was replaced by the corresponding sequence from DYRK1B. As shown in Fig. 5C, substitution of the CMGC insert in DYRK1B with that of DYRK1A (Chim3) enhanced tyrosine autophosphorylation by 3.7-fold. However, the relative phosphotyrosine level did not reach that of wild-type DYRK1A. Conversely, introducing the DYRK1B CMGC insert sequence into DYRK1A (Chim3i) only mildly affected its autophosphorylation. Notably, the enhanced autophosphorylation conferred by the DYRK1A-derived CMGC sequence was also observed when the chimera was expressed in *E. coli* (Fig. 5D). These findings indicate that differences within the CMGC insert sequence significantly contribute to the distinct autophosphorylation capacities of DYRK1A and DYRK1B in bacterial and cell-free expression systems.

### Functional interdependence of the proline mutations and the CMGC insert

Our results thus far reveal two key observations: first, the proline mutations (P332A, P333A) impair tyrosine autophosphorylation in DYRK1B but not in DYRK1A; and second, the CMGC insert plays a critical role in determining the distinct autophosphorylation capacities of these kinases. Based on this, we hypothesized that the differential effect of the proline mutations may be influenced by the sequence context provided by the adjacent CMGC insert.

**Fig. 6:**
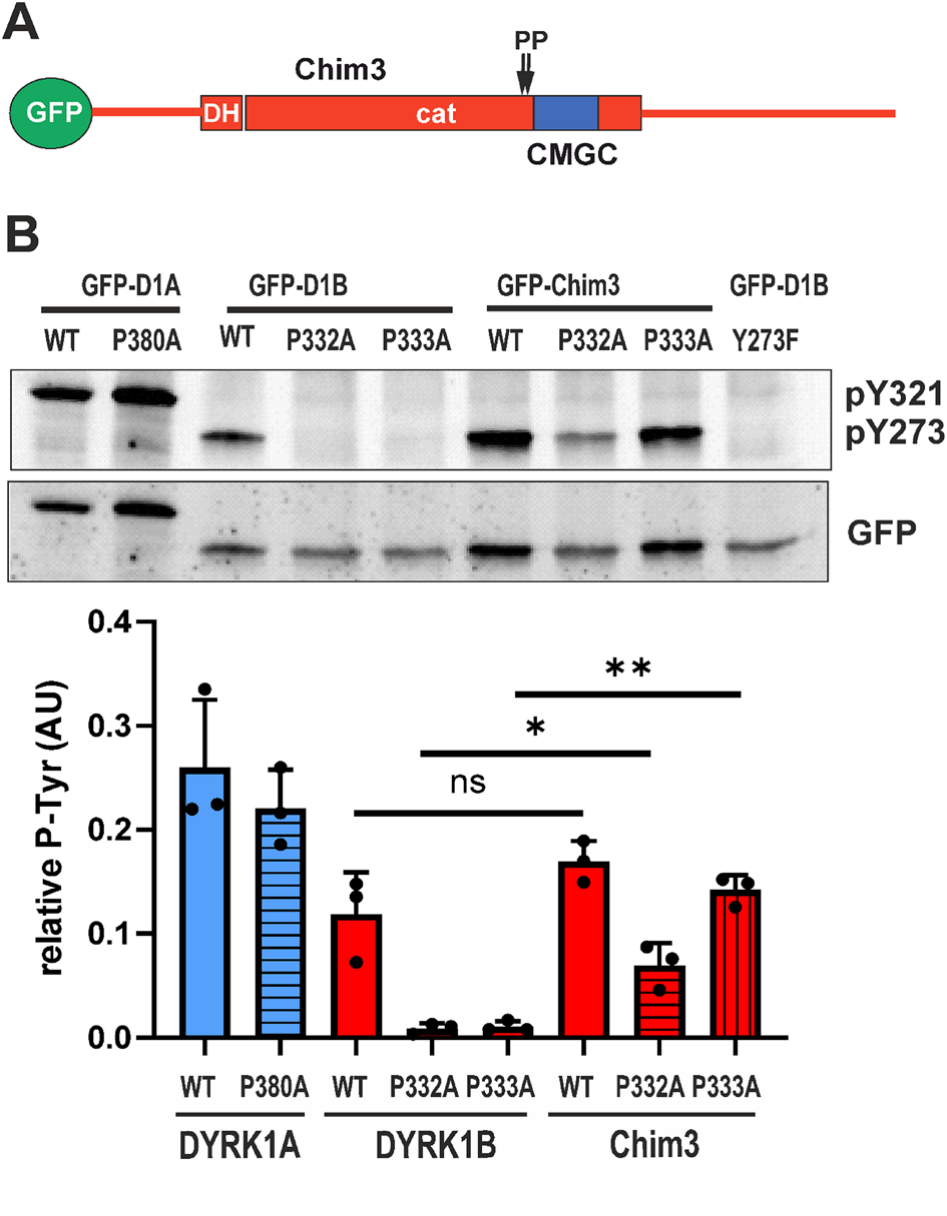
Analysis of the proline mutants in Chim3. (**A**) The scheme illustrates the position of the mutated prolines (P332A, P333A) in relation to the CMGC insert in the chimeric construct Chim 3. See Fig. 1A for the definition of the swapped sequences. (**B**) GFP-DYRK constructs were expressed in HEK293 cells, and total cellular lysates were subjected to immunoblot analysis. The graph summarizes the results from n = 3 experiments. Statistical significance was tested for the effect of the domain swap (DYRK1B vs. Chim3).

To examine this possibility, we introduced the P332A and P333A substitutions into the DYRK1B-based chimera Chim3, which harbors the CMGC insert from DYRK1A (Fig. 6A). Strikingly, the presence of the DYRK1A-derived CMGC insert substantially mitigated the detrimental effect of both proline mutations on Y273 autophosphorylation (Fig. 6B), although the defects were not fully rescued. These results indicate that the function of P332 and P333 in DYRK1B is, at least in part, dependent on the specific sequence of the CMGC insert, highlighting a functional interplay between these structural elements during kinase maturation.

### Structural modeling

**Fig. 7:**
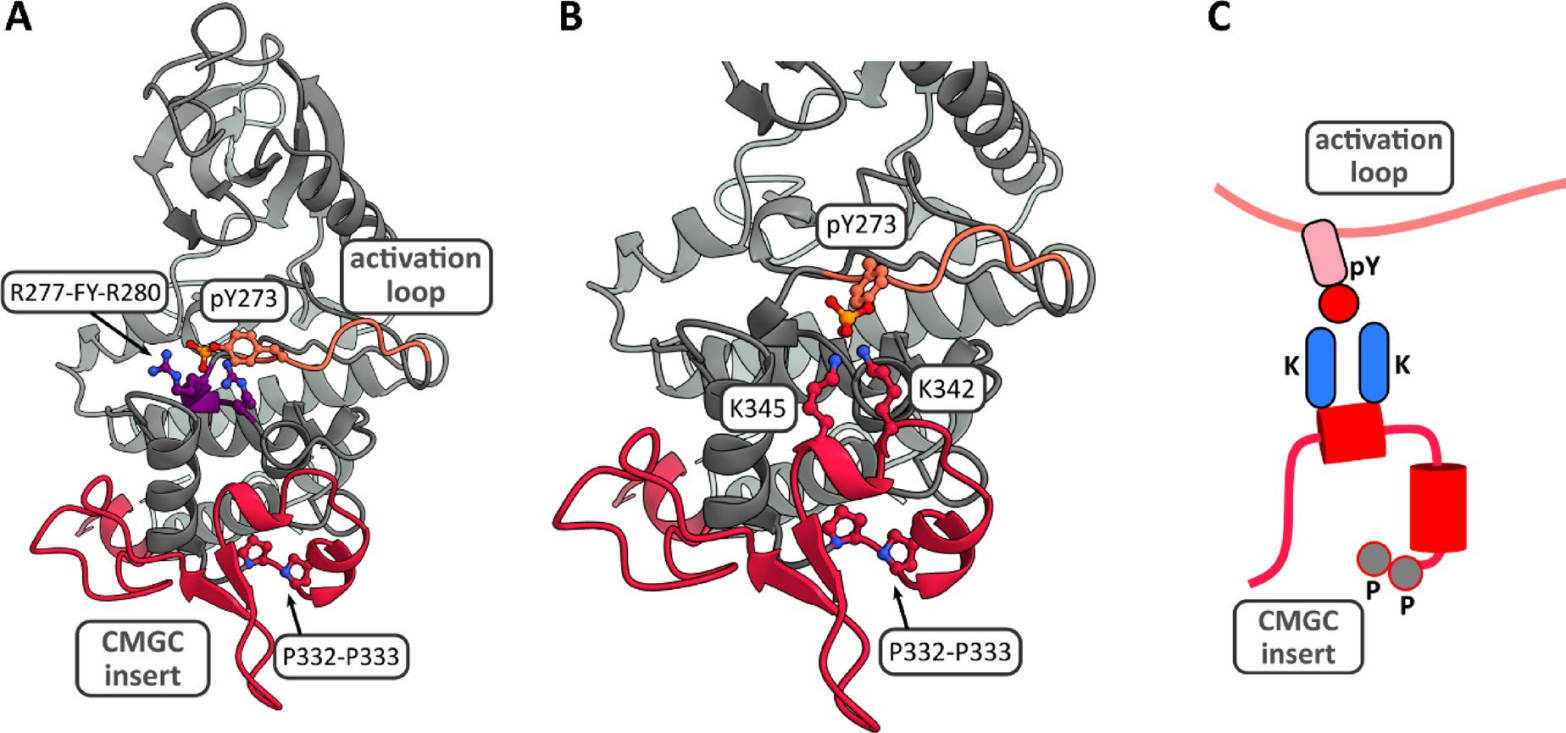
Potential interaction of the activation loop and the CMGC insert. (**A**) Ribbon structure of DYRK1B (8C2Z), highlighting the activation loop with pY273 and its salt-bridge interactions with R277 and R280 as well as the CMGC insert with the adjacent pair of prolines. (**C**) A modelled rotamer of pY273 in the DYRK1B structure (PDB: 8C2Z) shows the potential for an arrangement that forms putative salt bridges between the phosphate group and the two proximal lysine residues (K342 and K345) in the CMGC insert. The phosphotyrosine, arginines, lysines and the two proline residues (P332 and P333) are depicted as ball-and-stick representation. (**D**) Schematic representation of the hypothetical interaction between pY273 and the lysines in the CMGC insert. The cylinders represent two short alpha-helices of the CMGC insert.

The CMGC-specific insert is located between the αG and αH helices in the C-terminal lobe of the kinase domain and represents the most divergent region of the catalytic domain between DYRK1A and DYRK1B (Fig. 1A). In the 3D structure of the mature kinase fold, this region does not contribute to the core architecture of the kinase domain^27^ and does not make direct contact with the activation loop of DYRK1B (Fig. 7A). Interestingly, Kaltheuner and coworkers^28^ reported a crystal structure of HIPK3 in which the phosphotyrosine of the activation loop forms a salt bridge with R431 in the CMGC insert (PDB IDs: 7O7I, 7O7J, see supplementary Fig. S3). Given the similarity of the kinase domain fold in DYRKs and HIPKs, structural modelling was used to explore the rotameric states of the phosphorylated tyrosine residue (pY273) in the activation loop of DYRK1B. This revealed that a plausible side-chain orientation would enable electrostatic interactions between the phosphate moiety and two lysine residues (K342, K345) located within the CMGC insert (Fig. 7B, C). A similar interaction was also predicted for DYRK1A (supplementary Fig. S3). Although experimental evidence for this structure is lacking, these findings suggest that the CMGC insert could influence the activation-loop autophosphorylation of DYRK1 kinases.

### Characterization of a pathogenic substitution within the CMGC insert of DYRK1B

Folon et al.^29^ recently evaluated DYRK1B missense variants identified in a case–control cohort for obesity and type 2 diabetes. Six substitutions produced a complete loss-of-function phenotype (“P/LP-null”) in vitro, including R349W, which is located within the CMGC insert. Intriguingly, a second variant affecting the same residue—R349Q—was classified as functionally neutral in that study.

**Fig. 8:**
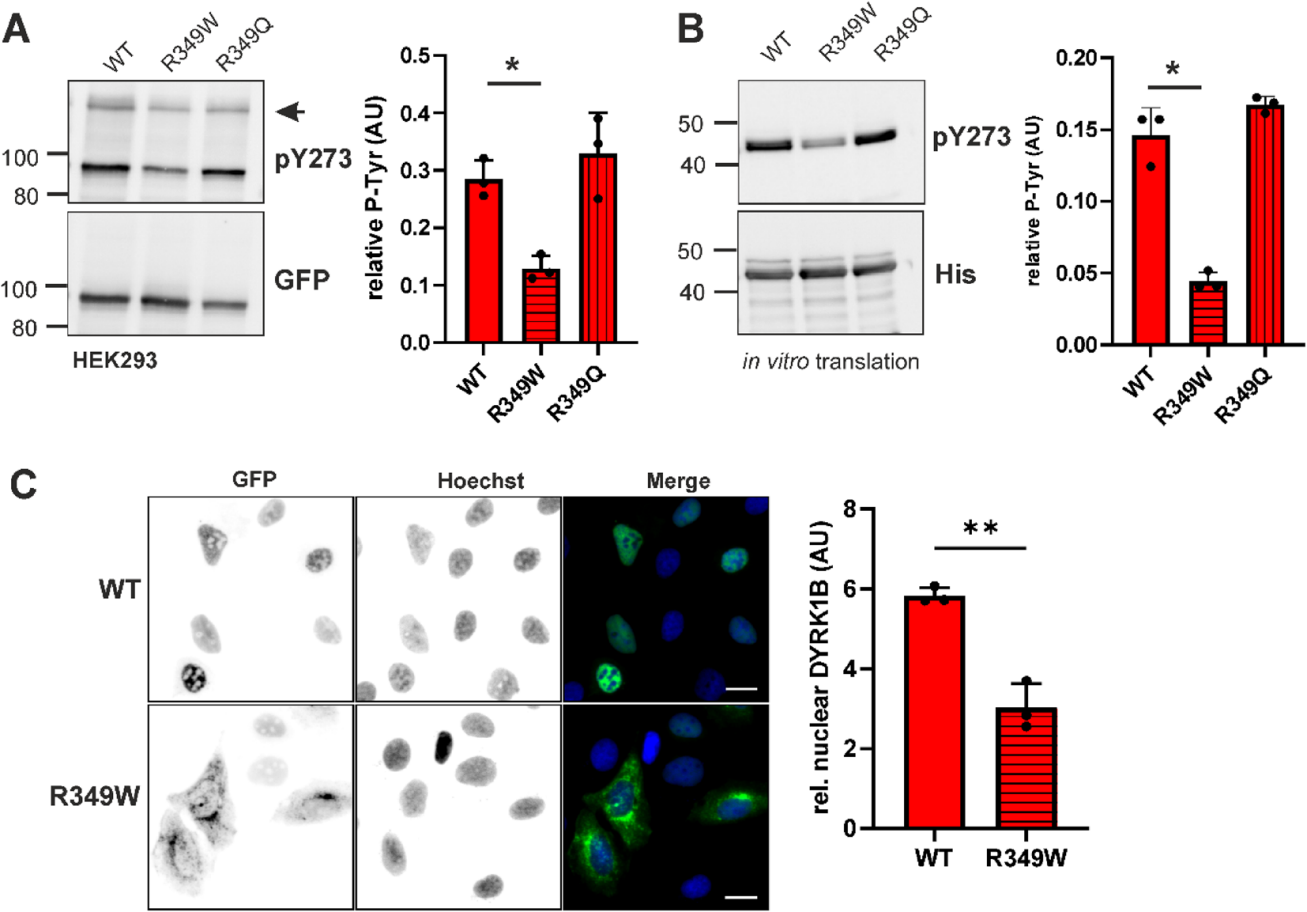
Effect of the pathogenic R349W missense mutation on DYRK1B autophosphorylation. (**A**, **B**) Expression of GFP-DYRK1B in HEK293 cells (**A**) and cell-free expression of His_6_-tagged DYRK1Bcat constructs (**B**). The blots illustrate representative experiments, and the graphs show the means and SD of n = 3 experiments. (**C**) Subcellular localization in U2OS cells. Representative fluorescence microscopy images (scale bars: 20 μm) and quantification of three independent experiments (mean ± SD) are shown.

Building on our evidence implicating the CMGC insert in DYRK1B folding and maturation, we compared the effects of R349W and R349Q on tyrosine autophosphorylation in both HEK293 cells and in an *E. coli*‑based cell‑free system (Fig. 8A, B). In each setting, substitution of R349 by the large hydrophobic tryptophan residue markedly reduced Y273 autophosphorylation, whereas the glutamine substitution (R349Q) had no significant impact. To further scrutinize the functional impact of the pathogenic R349W variant, we assessed its subcellular localization in U2OS cells (Fig. 8C). In this assay, the R349W substitution caused a substantial reduction of nuclear accumulation of GFP-DYRK1B. These findings indicate that precise sequence integrity within the CMGC insert is critical for DYRK1B maturation and suggest that disruptive mutations—exemplified by R349W—can drive clinically relevant DYRK1B dysfunction.

## Discussion

The activating autophosphorylation of DYRKs and other serine/threonine-specific protein kinases in the CMGC group is mechanistically distinct from the phosphorylation of exogenous substrates. The critical tyrosine in the activation loop does not conform to the kinase’s consensus recognition motif, nor is it readily positioned within the phosphoacceptor site of the catalytic cleft^5–7,30^. Consequently, tyrosine autophosphorylation by the nascent kinase requires unique structural adaptations and folding dynamics that differ from those involved in the phosphorylation of canonical substrate sites by the mature enzyme.

In this study, we leveraged the high sequence similarity between the DYRK1A and DYRK1B kinase domain in contrast to their strikingly different capacities for autonomous autophosphorylation to identify structural elements critical for kinase maturation. Our results demonstrate that sequence divergence within the CMGC insert accounts for key differences in autophosphorylation capacity between the two kinases. Specifically, the CMGC insert modulates the effect of mutations in the adjacent pair of conserved proline residues and influences the capacity to undergo autophosphorylation in bacterial expression systems. Consistent with the proposed role of the CMGC insert in domain folding, we found that the pathogenic R349W mutation—located far from the activation loop but introducing a bulky tryptophan residue into this region—impairs DYRK1B autophosphorylation and nuclear translocation. This observation supports the notion that the structural integrity of the CMGC insert is critical for the physiological function of human DYRK1B.

The CMGC insert—also referred to as the MAPK insert or CDK insert in the respective kinase families—is a flexible region located between α-helix G and α-helix H in the C-lobe of the catalytic domain. This structural element is the most structurally variable region of the catalytic domain among kinases within the CMGC branch of the kinome and is absent from other kinase families^27,28^. Notably, the CMGC insert does not contribute to the hydrophobic spine architecture that stabilizes the active conformation of the catalytic domain^31^, nor is it part of the three principal functional “sectors” of the kinase domain—those governing catalytic activity, substrate specificity, and regulatory input—as defined by Creixell et al.^32^.

Nevertheless, in CDKs and MAPKs, the CMGC insert serves regulatory purposes by providing a docking platform for signaling partners^27,33–36^. Interestingly, the CMGC insert of DYRK1A harbors a functional nuclear localization signal^37^. To our knowledge, no other regulatory function or protein–protein-interaction have been attributed to the CMGC insert in kinases that autoactivate through tyrosine autophosphorylation, such as DYRKs, HIPKs and GSK3.

Notably, the CMGC insert has previously been implicated in tyrosine autophosphorylation in a member of the MAPK family: unlike most other MAPKs, p38β is capable of intrinsic autophosphorylation. Using a chimeric strategy similar to our own, Beenstock and coworkers^38^ identified a minimal region of p38β that, when inserted into p38α, conferred MKK6-independent autoactivation. This 13-residue segment spans part of the G-helix and the MAPK insert and includes the conserved proline P242, which is structurally equivalent to P332 in DYRK1B. Together, these findings suggest that the CMGC insert, along with the adjacent proline residue(s), may serve an ancestral function in tyrosine autophosphorylation of CMGC kinases. Interestingly, the first proline is conserved across most CMGC kinase families, including the MAPKs, CDKs, and GSK3s, whereas the double-proline motif is unique to DYRKs (see supplementary Fig. S4 for an alignment of all human CMGC kinases).

Our results support the finding of Lee et al.^17^ that P332 is required for tyrosine autophosphorylation of DYRK1B in mammalian cells. However, this proline residue is not strictly essential for DYRK1B maturation, as the P332A mutation impairs autophosphorylation in *E. coli* only when the kinase is expressed at 37 °C, but not at 19 °C. This temperature-dependent effect suggests that the mutation disrupts proper folding of DYRK1B in a thermosensitive manner. Notably, the deleterious impact of the P332A mutation persists even at a reduced temperature (20 °C) when the catalytic domain alone is expressed in vitro (Fig. 4B). This observation implies that the folding defect observed in cell-free expression is partially compensated in the *E. coli* expression system by factors such as bacterial chaperones^13^, the presence of the N-terminal region, or the N-terminal GST fusion tag, which slows translation and may thereby facilitate proper folding. It is conceivable that the prolines themselves promote correct folding of the catalytic domain by transiently delaying the process through the intrinsically slow *cis–trans* isomerization of their peptide bonds, thereby reducing the formation of misfolded or abortive folding intermediates^39^.

In contrast to the findings of Lee et al.^17^, we consistently observed across all experimental systems—mammalian cells, *E. coli*, and cell-free expression—that the P333A mutation had deleterious effects comparable to those of the neighboring P332A mutation. Some assays showed minimal residual activity of the P333A mutant (Figs. 1B, 4A). Substantial differences between the two mutations were detected in the SF3B1 phosphorylation assay, where P333A retained residual catalytic activity (Fig. 1F), and in the analysis of the Chimera3 construct, where P333A did not significantly impair tyrosine autophosphorylation (Fig. 6B). These results indicate that the relative impact of the individual proline mutations depends on the surrounding sequence context—specifically, the sequence of the adjacent CMGC insert—with the P333A substitution exerting a milder effect. Nonetheless, both proline residues are essential for the correct folding and activation of the DYRK1B catalytic domain.

In contrast to our findings for DYRK1B, mutation of the first proline (P380) in DYRK1A did not impair tyrosine autophosphorylation or alter the subcellular localization of DYRK1A in mammalian cells. A similar divergence was observed across species: while the homologous proline mutation abolished tyrosine autophosphorylation in *Xenopus* DYRK1B, the corresponding mutation in zebrafish DYRK1B had no apparent effect. Notably, our previous data^15^ showed that zebrafish DYRK1B—unlike its *Xenopus* ortholog—autophosphorylates as efficiently as mammalian DYRK1A in a cell-free in vitro translation system. Despite this, the proline mutation still reduced phosphotyrosine levels of human DYRK1A and zebrafish DYRK1B in the cell-free system, suggesting a latent requirement for this residue in kinase maturation that is compensated in mammalian cells. Importantly, our findings challenge the hypothesis that this proline participates in a general regulatory mechanism shared among CMGC kinases, as proposed by Lee et al*.*^17^.

**Fig. 9:**
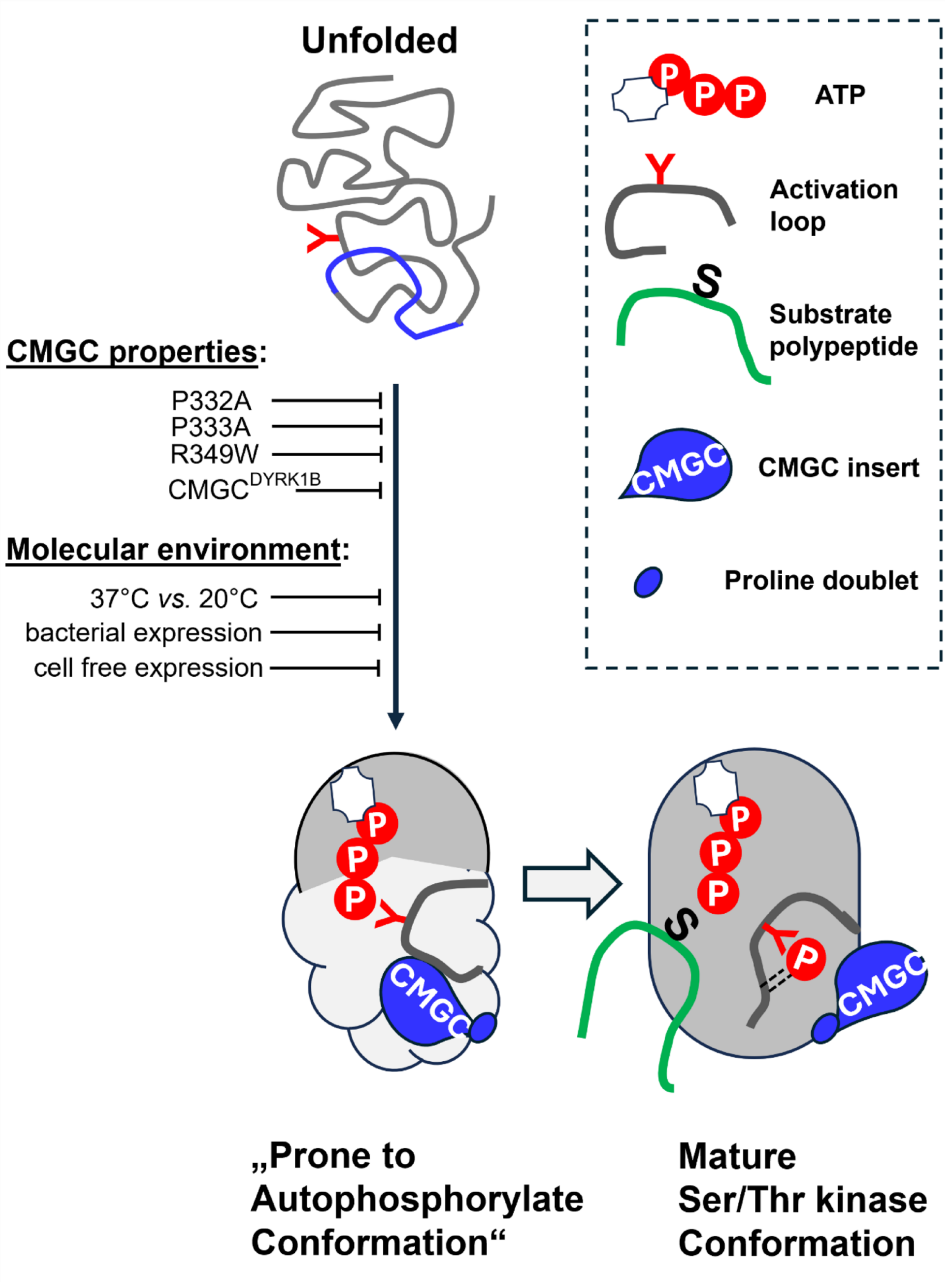
Role of the CMGC insert in the folding and maturation of the DYRK1 kinase domain. Cartoon illustration of DYRK1 domain folding and activation. Proper folding is influenced by the sequence of the CMGC insert and the cellular environment. During maturation, the kinase adopts a flexible, partially folded conformation that allows intramolecular *cis*-autophosphorylation of the conserved activation loop tyrosine (indicated by the red Y), a prerequisite for acquiring full catalytic activity as a serine/threonine kinase.

Given the markedly different capacities for autonomous tyrosine autophosphorylation between mammalian DYRK1A and DYRK1B, we hypothesize that the penetrance of the proline mutation is context-dependent and shaped by the thermodynamic and chaperone environment during folding (Fig. 9). Lower expression temperatures improve folding outcomes relative to higher temperatures, and the CMGC insert of DYRK1A appears to provide a more stabilizing structural context than the corresponding sequence in DYRK1B. Furthermore, expression in mammalian cells—where the CDC37-HSP90 chaperone system supports kinase maturation—provides a more permissive folding environment than bacterial expression in *E. coli*, which in turn is more favorable than cell-free systems. Notably, the bacterial HSP70 chaperone DnaK has been shown to promote DYRK1A autophosphorylation in a cell free expression system^13^.

Lee et al. proposed that hydroxylation of P332 in DYRK1B and P380 in DYRK1A by the prolyl hydroxylase PHD1 is required for their activation, providing an additional layer of control in cancer stem cells^17^. This mechanism appears to be restricted to physiological expression in eukaryotic cells, since active DYRK1B can be readily expressed in *E. coli*, which lacks prolyl-4-hydroxylase activity^40^, and even in cell-free systems. This discrepancy suggests that proline hydroxylation is not strictly essential for autophosphorylation. Nevertheless, it remains possible that hydroxylation promotes the adoption of a “prone to autophosphorylate conformation” by displacing an endogenous allosteric inhibitor that may be present in mammalian cells but absent from bacterial or in vitro systems^17^. In our experiments, pharmacological inhibition of prolyl hydroxylase activity with Roxadustat did not alter the phosphotyrosine levels of DYRK1B in HEK293 cells. While the strong induction of HIF1α validated PHD inhibition, we cannot exclude the possibility that inhibition was incomplete and therefore insufficient to block DYRK maturation (see also CoCl₂ experiments).

Finally, it is noteworthy that one of the six DYRK1B variants associated with monogenic obesity and type 2 diabetes, as identified by Folon and co-workers^29^, affects the CMGC insert (R349W). Disease-causing single nucleotide polymorphisms (SNPs) rarely localize to the C-terminal subdomains of the kinase domain, which are not directly involved in catalysis or the structural integrity of the kinase fold^41^. The R349W variant was classified as a complete loss-of-function allele due to its fully inhibitory effect on Wnt signaling^29^. In our study, we observed a marked reduction in tyrosine autophosphorylation and reduced nuclear translocation of this variant, further underscoring the functional relevance of the CMGC insert in the maturation process.

## Materials and methods

### Antibodies

Antibodies are listed in supplementary Table S2.

### Cloning of expression plasmids and mutagenesis

Expression plasmids for wild type proteins and deletion constructs are listed in supplementary Table S1. Chimeric DYRK1A-DYRK1B cDNAs were planned with the help of the NEBuilder Assembly Tool and cloned using the NEBuilder HiFi DNA Assembly Cloning Kit and Q5 High-Fidelity DNA Polymerase (#E5520S and #M0491S, New England Biolabs, Inc., Ipswich, MA, USA). Point mutations were introduced using the QuikChange method. All cDNAs were sequenced to verify the fidelity of the PCR.

### Mammalian expression and immunoprecipitation

HEK293tsa201 cells (Sigma-Aldrich # 96121229, RRID:CVCL_2737) and U2OS Flip-In cells (RRID:CVCL_0042, kind gift of Haico van Attikum^42^) were cultured in DMEM/F12 medium supplemented with 10% fetal bovine serum at 37 °C in a humidified atmosphere containing 5% CO₂. HeLa cells (RRID:CVCL_0030) were maintained in RPMI medium under the same conditions. Transient transfections were performed in 6-well plates using 1 µg DNA per well and FuGENE HD Transfection Reagent (Promega Corporation, Madison, WI, USA). For preparation of total cell lysates, cells were lysed in 100 µL denaturing lysis buffer (20 mM Tris–HCl, pH 7.5; 1% SDS) per well at 96 °C and sonicated.

For immunoprecipitation experiments (Fig. 1C), HeLa cells were seeded in 6-cm dishes and transfected with 2 µg DNA per dish. Cells were lysed in native lysis buffer (50 mM Tris–HCl, pH 7.5; 150 mM NaCl; 1 mM EDTA; 1 mM NaF; 0.5% Igepal) supplemented with protease and phosphatase inhibitors. GFP-DYRK1 fusion proteins were immunoprecipitated using GFP-Trap magnetic beads (ChromoTek, Planegg-Martinsried, Germany).

For SF3B1 phosphorylation assays, HEK293tsa201 cells were seeded in 6-well plates and sequentially transfected with expression plasmids encoding GFP-SF3B1-NT (500 ng/well) and the indicated GFP-DYRK1B constructs (50 ng/well). Cells were lysed in denaturing lysis buffer, and phosphorylation of SF3B1 at Thr434 was analyzed by immunoblotting using a custom phospho-specific antibody^23^.

### Bacterial expression

GST fusion proteins were expressed in Novagen *E. coli* BL21 Rosetta cells and partially purified by affinity adsorption to glutathione sepharose CL-4B as described previously^15^. For immunoblot analysis, bacteria were grown at either 37 °C or 19 °C as indicated, and bound GST fusion proteins were eluted under denaturing conditions (Laemmli’s sample buffer supplemented with 200 mM DTT, 96 °C). The amounts of protein subjected to SDS-PAGE were adjusted according to Coomassie staining intensities. For assays of catalytic activity (Fig. S1C), GST-DYRK1A (expressed at 37 °C) and GST-DYRK1B (expressed at 8 °C) were eluted under native conditions using 50 mM Tris–HCl pH 8.0, 10 mM reduced glutathione and stored at − 80 °C until use.

### Cell free expression

In vitro translation of DYRK1 constructs was performed with the NEBExpress Cell-free Protein Synthesis System based on *E. coli* extracts (#E5360S, New England Biolabs, Ipswich, MA, USA). Reactions were performed in a volume of 6 µL with 40 ng plasmid DNA and 0.12 μL murine RNase-Inhibitor (#M0314S, New England Biolabs, Ipswich, MA, USA) in a total volume of 6 μL. Reactions were stopped by adding Laemmli’s sample buffer supplemented with DTT (200 mM) and incubation at 96 °C. Samples were stored at − 20 °C before SDS-PAGE and Western blot analysis.

### Immunoblot analysis

Protein samples were separated by SDS–PAGE and transferred to nitrocellulose membranes (0.45 μm; Amersham Protran, GE Healthcare Life Sciences, Marlborough, MA, USA; #1060002). Membranes were blocked with 3% bovine serum albumin (BSA) in TBS-T buffer (50 mM Tris–HCl, pH 7.5; 150 mM NaCl; 0.1% Tween-20) and incubated overnight at 4 °C with primary antibodies. After washing, membranes were incubated for 1 h at room temperature with HRP-conjugated secondary antibodies. Chemiluminescence signals were detected using the LAS-3000 imaging system (Fujifilm, Düsseldorf, Germany), and band intensities were quantified with Multi Gauge Analysis Software (Fujifilm). Relative phosphotyrosine levels were calculated as the ratio of the p-Tyr signal to total protein (based on the intensity of GFP, GST, or His_6_ bands). To normalize the data obtained in replica experiments, relative phosphotyrosine values for each sample were normalized to the sum of all relevant data points from one blot^43^. Uncropped images of the blots are provided as supplementary information.

### Fluorescence microscopy, image processing and analysis

U2OS Flp/In cells were seeded at a density of 30.000 cells per well in a 24-well plate and transiently transfected with GFP-DYRK constructs (50 ng/well). After 24 h, cells were fixed with 3.7% paraformaldehyde for 10 min at room temperature. After washing with PBS, cells were quenched with 100 mM glycine in PBS for 20 min and then permeabilized by incubation in PBS containing 0.1% Triton-X and 1% BSA for 10 min. After three washing steps, nuclei staining was performed with Hoechst 33,342 (2 µg/mL) for 10 min at RT and samples were stored in PBS at 4 °C until imaging.

Fluorescence microscopy was performed on a Leica DMi8 Inverted widefield microscope. (20 × objective). Five images per condition were analyzed to evaluate at least 60 cells per data point. Image processing was conducted with the imaging processing software ImageJ/Fiji 1.54p. Nuclear-to-cytoplasmic (N/C) quantification was performed with a customized N/C Quantification macro^44^, available at https://github.com/NickCondon/Nuclei-Cyto_MeasuringScipt) with minor adjustments. Briefly, nuclear masks were created from Hoechst staining, and individual nuclei were defined as ROI for measuring nucleus mean fluorescence intensity (MFI). Cytoplasm MFI was determined by expanding the nuclei ROI and creating 1.5 pixel-wide ring to capture the adjacent cytoplasmic area.

### Structural modelling

Structural models of the proteins were obtained from the Protein Data Bank (PDB). DYRK1A (PDB ID: 9FPX), DYRK1B (PDB ID: 8C2Z) and HIPK3 (PDB ID: 7O7J). The structures were loaded into ChimeraX (version 1.10, 2025-06-26) and visualised using cartoon and atomic representations. Structural manipulations, including side-chain rotamer modelling and selection, were performed using the built-in ChimeraX tools.

### Statistics

Statistical significance of differences between WT kinases and point mutants thereof or between WT and chimeric constructs were tested by t test. (*p < 0.05, **p < 0.01).

## Supplementary Information

Below is the link to the electronic supplementary material.

Supplementary Material 1

## Data Availability

All relevant data of this study are available within the article and its supplementary information files.

## Abbreviations

CMGC: A group of protein kinases named after the initials of its main kinase families (CDKs, MAPKs, GSKs, CLKs)
GFP: Green fluorescent protein
GST: Glutathione S-transferase
PHD: Prolyl hydroxylase domain protein
