# Supplementary material for: Critical role of the CMGC insert sequence for tyrosine autophosphorylation in the protein kinase DYRK1B
Source: Sci Rep. 2025 Dec 24;15:44423. doi: 10.1038/s41598-025-33562-x (PMC12738607; doi:10.1038/s41598-025-33562-x)

Supplementary Information

Silvia Detro-Dassen, Katharina Schwandt, Philip Helmich, Stefan Düsterhöft, Walter Becker

Critical Role of the CMGC Insert Sequence for Tyrosine Autophosphorylation in the Protein Kinase DYRK1B

Corresponding Author

Walter Becker – *Institute of Pharmacology and Toxicology, RWTH Aachen University, 52074 Aachen, Germany;* Email: wbecker@ukaachen.de

Content:

Supplementary Figures **2**

Fig. S1. Effect of $CoCl_2$ on DYRK1B 2

Fig. S2: Temperature-dependent effect of proline mutations on GST-DYRK1B 3

Fig. S3: Hypothetical interaction of the phosphotyrosine in the activation loop with the CMGC insert 4

Fig. S4: Conservation of the proline motif in human CMGC kinases 5

Key Resources

Table S1: Expression plasmids 6

Table S2: Antibodies 7

Uncropped Western blots 8

Supplementary Figures

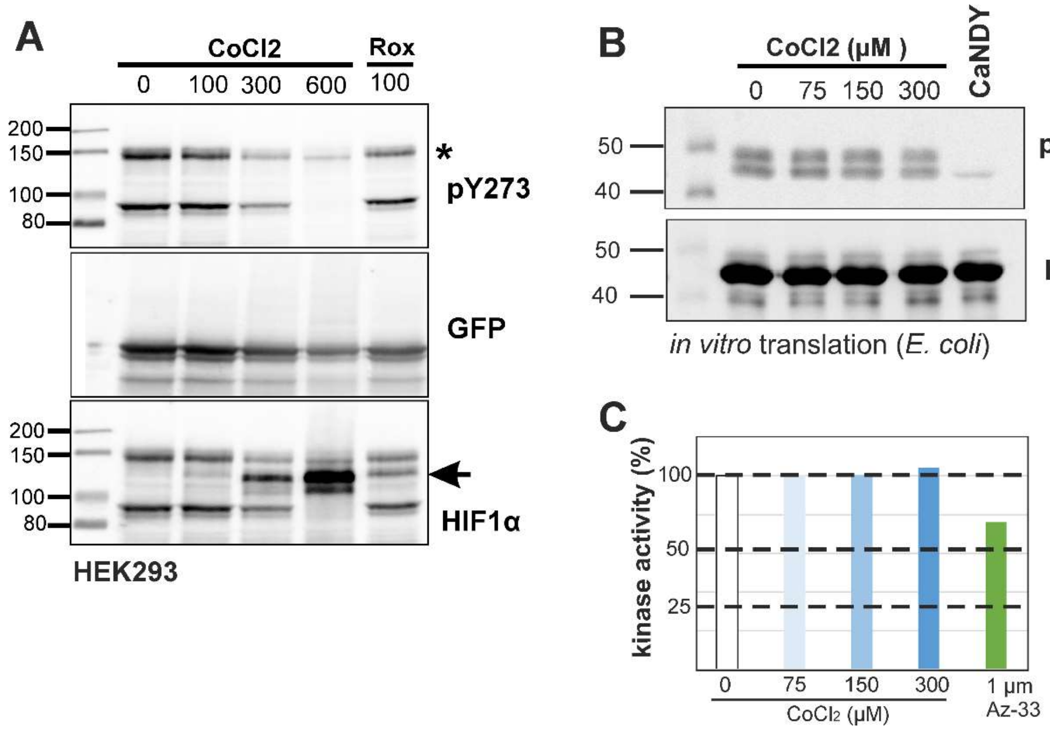

Fig. S1. Effect of $CoCl_2$ on DYRK1B

A, Effect of $CoCl_2$ in HEK293 cells (supporting Fig. 1E in the main manuscript). Five hours after transient transfection, cells were treated with $CoCl_2$ or Roxadustat as indicated. HIF1α induction was much higher at 600 µM $CoCl_2$ than 300 µM, indicating that PHD activity is only partially inhibited at lower $CoCl_2$ concentrations or at 100 µM Roxadustat. However, 600 µM $CoCl_2$ abolished expression of GFP-DYRK1B. The asterisks marks the endogenous pY361-HIPK2 band.
B, CoCl2 does not inhibit DYRK1B tyrosine autophosphorylation in a cell-free system. CaNDY, a small-molecule DYRK1 inhibitor (Sonamoto et al 2015), was used a positive control.
C, $CoCl_2$ does not inhibit catalytic activity of mature DYRK1B. Recombinant GST-DYRK1B was subjected to a kinase assay (Kinase-Glo Assay, Promega) with a peptide substrate (DYRKtide). In this assay, kinase activity is quantified based on ATP consumption. Owing to the non-linear nature of the assay, results are shown in comparison with reactions containing half (50%) or a quarter (25%) of the kinase. AZ-33, a small molecule DYRK1B inhibitor (Kettle et al. 2015), was included as a positive control.

Kettle JG, Ballard P, Bardelle C, Cockerill M, Colclough N, Critchlow SE, Debreczeni J, Fairley G, Fillery S, Graham MA, Goodwin L, Guichard S, Hudson K, Ward RA, Whittaker D. Discovery and optimization of a novel series of Dyrk1B kinase inhibitors to explore a MEK resistance hypothesis. *J Med Chem.* 58:2834-44 (2015).

Sonamoto R, Kii I, Koike Y, Sumida Y, Kato-Sumida T, Okuno Y, Hosoya T, Hagiwara M. Identification of a DYRK1A Inhibitor that Induces Degradation of the Target Kinase using Co-chaperone CDC37 fused with Luciferase nanoKAZ. *Sci Rep.* 5:12728 (2015).

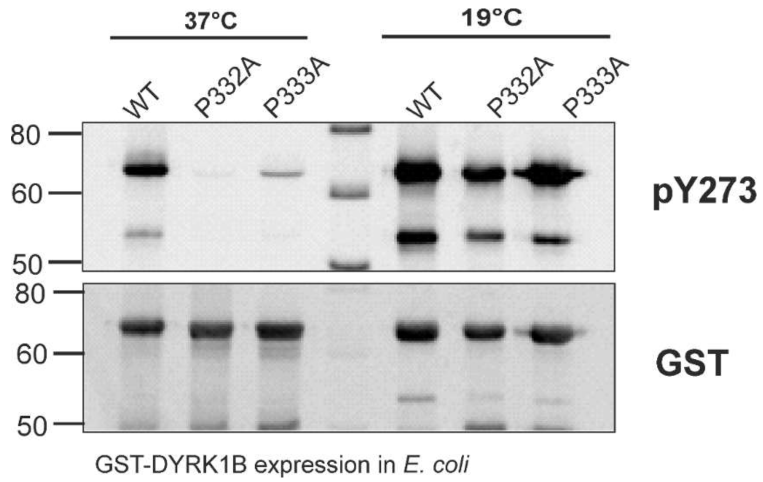

Figure S2: Temperature-dependent effect of proline mutations on GST-DYRK1B tyrosine autophosphorylation (supporting Fig. 4A in the main manuscript).
The experiment shown in Fig. 4A was reproduced with independent preparations of the GST fusion proteins.

A

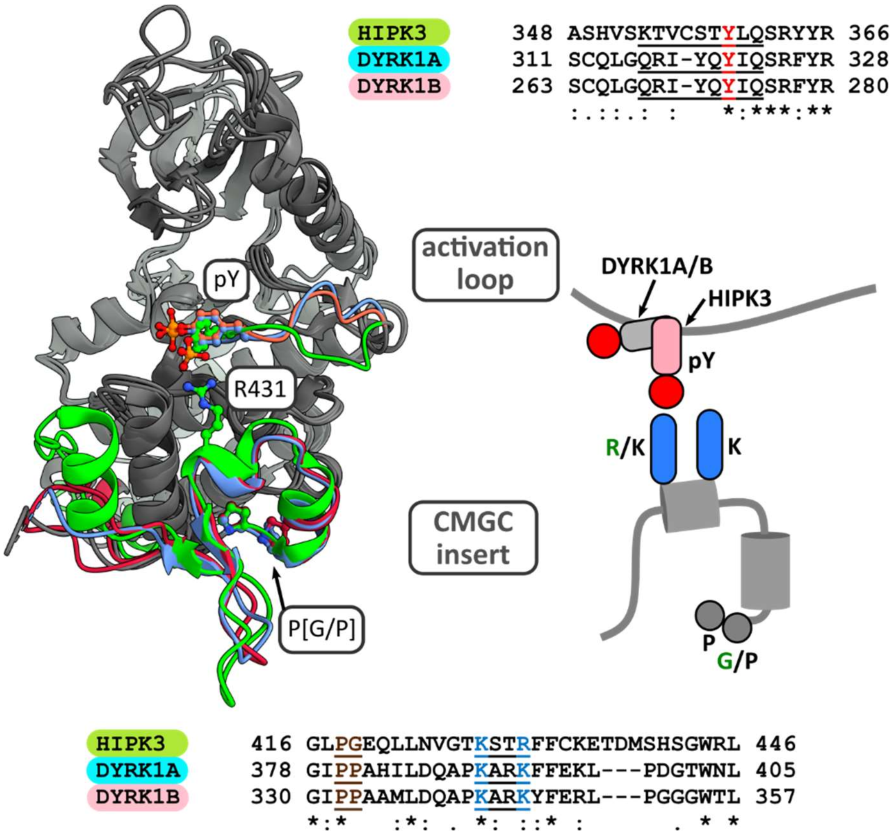

B

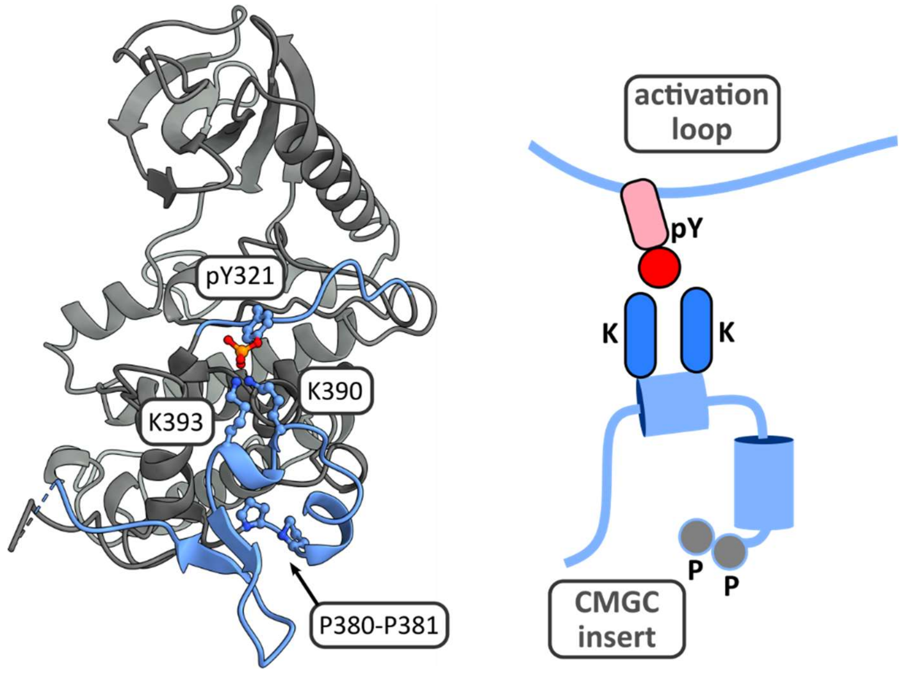

Figure S3: Hypothetical interaction of the phosphotyrosine in the activation loop with the CMGC insert (supporting Fig. 7 in the main manuscript)

A, The ribbon diagram overlay of DYRK1A (PDB ID: 9FPX), DYRK1B (PDB ID: 8C2Z), and HIPK3 (PDB ID: 7O7J) structures illustrates the overall similarity of the fold with the divergent orientation of the activation loop phosphotyrosine (pY).

B, A modelled rotamer of pY321 in the DYRK1A structure shows possible salt bridges between the phosphate group and the two lysine residues (K342 and K345) in the CMGC insert.

```
                        ≈≈≈≈≈αG≈≈≈≈≈≈  ↓↓ -------- CMGC insert------------
CMGC_DYRK1A             ANEVDQMNKIVEVLGIPPAHILDQAPKARKFFEKLPDGTWNLKKTKDGKR
CMGC_DYRK1B             SNEVDQMNRIVEVLGIPPAAMLDQAPKARKYFERLPGGGWTLRRTKELRK
CMGC_DYRK2              EDEGDQLACMIELLGMPSQKLLDASKRAKNFVSSKGYPRYCTVTTLSDGS
CMGC_DYRK3              EDEGDQLACMMELLGMPPPKLLEQSKRAKYFINSKGIPRYCSVTTQADGR
CMGC_DYRK4              ENEVEQLACIMEVLGLPPAGFIQTASRRQTFFDSKGFPKNITNNRGKKRY
CMGC_PRPF4B             KTNNHMLKLAMDLKGKMPNKMIRKGVFKDQHFDQNLNFMYIEVDKVTERE
CMGC_HIPK1              ASEYDQIRYISQTQGLPAEYLLSAGTKTTRFFNRDPNLGYPLWRLKTPEE
CMGC_HIPK2              ASEYDQIRYISQTQGLPAEYLLSAGTKTTRFFNRDTDSPYPLWRLKTPDD
CMGC_HIPK3              ALEYDQIRYISQTQGLPGEQLLNVGTKSTRFFCKETDMSHSGWRLKTLEE
CMGC_HIPK4              NNEYDQVRYICETQGLPKPHLLHAACKAHHFFKRNPHPDAANPWQLKSSA
CMGC_CLK1               HDSKEHLAMMERILGPLPKHMIQKTRKRKYFHHDRLDWDEHSSAGRYVSR
CMGC_CLK2               HDNREHLAMMERILGPIPSRMIRKTRKQKYFYRGRLDWDENTSAGRYVRE
CMGC_CLK3               HENREHLVMMEKILGPIPSHMIHRTRKQKYFYKGGLVWDENSSDGRYVKE
CMGC_CLK4               HDSKEHLAMMERILGPIPQHMIQKTRKRKYFHHNQLDWDEHSSAGRYVRR
CMGC_SRPK1              TRDEDHIALIIELLGKVPRKLIVAGKYSKEFFTKKGDLKHITKLKPWGLF
CMGC_SRPK2              SRDEDHIAHIIELLGSIPRHFALSGKYSREFFNRRGELRHITKLKPWSLF
CMGC_SRPK3              SRDEDHIAHIVELLGDIPPAFALSGRYSREFFNRRGELRHIHNLKHWGLY
CMGC_CSNK2A1            HDNYDQLVRIAKVLGTEDLYDYIDKYNIELDPRFNDILGRHSRKRWERFV
CMGC_CSNK2A2            QDNYDQLVRIAKVLGTEELYGYLKKYHIDLDPHFNDILGQHSRKRWENFI
CMGC_CSNK2A3            RDNYDQLVRIAKFLGTEDLYGYIDKYNIELDPRFNDILGRHSRKRWERFV
CMGC_GSK3A              DSGVDQLVEIIKVLGTPTREQIREMNPNYTEFKFPQIKAHPWTKVFKSRT
CMGC_GSK3B              DSGVDQLVEIIKVLGTPTREQIREMNPNYTEFKFPQIKAHPWTKVFRPRT
CMGC_ICK                ASEIDTIFKICQVLGTPKKTDWPEGYQLSSAMNFRWPQCVPNNLKTLIPN
CMGC_MAK                TSEVDEIFKICQVLGTPKKSDWPEGYQLASSMNFRFPQCVPINLKTLIPN
CMGC_MOK                VNELDQISKIHDVIGTPAQKILTKFKQSRAMNFDFPFKKGSGIPLLTTNL
CMGC_CDKL1              KSDVDQLYLIRKTLGDLIPRHQQVFSTNQYFSGVKIPDPEDMEPLELKFP
CMGC_CDKL2              DSDIDQLYHIMMCLGNLIPRHQELFNKNPVFAGVRLPEIKEREPLERRYP
CMGC_CDKL3              SSDLDLLHKIVLKVGNLSPHLQNIFSKSPIFAGVVLPQVQHPKNARKKYP
CMGC_CDKL4              KSDVDQLYLIIRTLGKLIPRHQSIFKSNGFFHGISIPEPEDMETLEEKFS
CMGC_CDKL5              ESEIDQLFTIQKVLGPLPSEQMKLFYSNPRFHGLRFPAVNHPQSLERRYL
CMGC_MAPK8 (JNK1)       RDYIDQWNKVIEQLGTPCPEFMKKLQPTVRTYVENRPKYAGYSFEKLFPD
CMGC_MAPK9 (JNK2)       TDHIDQWNKVIEQLGTPSAEFMKKLQPTVRNYVENRPKYPGIKFEELFPD
CMGC_MAPK10 (JNK3)      RDYIDQWNKVIEQLGTPCPEFMKKLQPTVRNYVENRPKYAGLTFPKLFPD
CMGC_MAPK11 (p38β)      SDYIDQLKRIMEVVGTPSPEVLAKISSEHARTYIQSLPPMPQKDLSSIFR
CMGC_MAPK12 (p38γ)      SDHLDQLKEIMKVTGTPPAEFVQRLQSDEAKNYMKGLPELEKKDFASILT
CMGC_MAPK13 (p38δ)      KDYLDQLTQILKVTGVPGTEFVQKLNDKAAKSYIQSLPQTPRKDFTQLFP
CMGC_MAPK14 (p38α)      TDHIDQLKLILRLVGTPGAELLKKISSESARNYIQSLTQMPKMNFANVFI
CMGC_MAPK3 (ERK1)       KHYLDQLNHILGILGSPSQEDLNCIINMKARNYLQSLPSKTKVAWAKLFP
CMGC_MAPK1 (ERK2)       KHYLDQLNHILGILGSPSQEDLNCIINLKARNYLLSLPHKNKVPWNRLFP
CMGC_MAPK7 (ERK5)       KNYVHQLQLIMMVLGTPSPAVIQAVGAERVRAYIQSLPPRQPVPWETVYP
CMGC_MAPK15 (ERK7)      TSTLHQLELILETIPPPSEEDLLALGSGCRASVLHQLGSRPRQTLDALLP
CMGC_MAPK4              AHELEQMQLILETIPVIREEDKDELLRVMPSFVSSTWEVKRPLRKLLPEV
CMGC_MAPK6              AHELEQMQLILESIPVVHEEDRQELLSVIPVYIRNDMTEPHKPLTQLLPG
CMGC_NLK                QSPIQQLDLITDLLGTPSLEAMRTACEGAKAHILRGPHKQPSLPVLYTLS
CMGC_CDK1               DSEIDQLFRIFRALGTPNNEVWPEVESLQDYKNTFPKWKPGSLASHVKNL
CMGC_CDK10              TSEIHQIDLIVQLLGTPSENIWPGFSKLPLVGQYSLRKQPYNNLKHKFPW
CMGC_CDK11A             NSEIDQINKVFKELGTPSEKIWPGYSELPVVKKMTFSEHPYNNLRKRFGA
CMGC_CDK11B             KSEIDQINKVFKDLGTPSEKIWPGYSELPAVKKMTFSEHPYNNLRKRFGA
CMGC_CDK12              NLELAQLELISRLCGSPCPAVWPDVIKLPYFNTMKPKKQYRRRLREEFSF
CMGC_CDK13              NQELAQLELISRICGSPCPAVWPDVIKLPYFNTMKPKKQYRRKLREEFVF
CMGC_CDK14              KDIQDQLERIFLVLGTPNEDTWPGVHSLPHFKPERFTLYSSKNLRQAWNK
CMGC_CDK15              SNILEQLEKIWEVLGVPTEDTWPGVSKLPNYNPEWFPLPTPRSLHVVWNR
CMGC_CDK16              STVEEQLHFIFRILGTPTEETWPGILSNEEFKTYNYPKYRAEALLSHAPR
CMGC_CDK17              STVEDELHLIFRLLGTPSQETWPGISSNEEFKNYNFPKYKPQPLINHAPR
CMGC_CDK18              STVKEELHLIFRLLGTPTEETWPGVTAFSEFRTYSFPCYLPQPLINHAPR
CMGC_CDK19              PFHHDQLDRIFSVMGFPADKDWEDIRKMPEYPTLQKDFRRTTYANSSLIK
CMGC_CDK2               DSEIDQLFRIFRTLGTPDEVVWPGVTSMPDYKP-SFPKWARQDFSKVVPP
CMGC_CDK20              KNDIEQLCYVLRILGTPNPQVWPELTELPDYNKISFKEQVPMPLEEVLPD
CMGC_CDK3               DSEIDQLFRIFRMLGTPSEDTWPGVTQLPDYKG-SFPKWTRKGLEEIVPN
CMGC_CDK4               NSEADQLGKIFDLIGLPPEDDWPRDVSLPRGAFPPRGPRPVQSVVPEME-
CMGC_CDK5               NDVDDQLKRIFRLLGTPTEEQWPSMTKLPDYKPYPMYPATTSLVNVVPKL
CMGC_CDK6               SSDVDQLGKILDVIGLPGEEDWPRDVALPRQAFHSKSAQPIEKFVTDID-
CMGC_CDK7               DSDLDQLTRIFETLGTPTEEQWPDMCSLPDYVTFKSFPGIPLHHIFSAAG
CMGC_CDK8               PYHHDQLDRIFNVMGFPADKDWEDIKKMPEHSTLMKDFRRNTYTNCSLIK
CMGC_CDK9               NTEQHQLALISQLCGSITPEVWPNVDNYELYEKLELVKGQKRKVKDRLKA
```

Figure S4: Conservation of the proline motif in human CMGC kinases.

The prolines (highlighted in bold print) lie just after the αG helix at the N-terminal end of the CMGC insert.

The alignment was adopted from Modi V, Dunbrack RL. Structurally-Validated Multiple Sequence Alignment of 497 Human Protein Kinase Domains. *Sci Rep* **9:19790** (2019) https://dunbrack.fccc.edu/kincore/alignment .

Key Resources

Table S1: Expression plasmids for wild type proteins and deletion constructs

expressed protein	Species	NCBI Refseq accession number	Vector	Source
GFP-rDYRK1A (full length)	*Rattus norvegicus*	NP_036923.1	pEGFP-C1	Becker et al. 1998
GFP-hDYRK1B (full length)	*Homo sapiens*	NP_004705.1	pEGFP-C1	Leder et al. 1999
GFP-zDYRK1B (full length)	*Danio rerio*	XP_005158272.1	pEGFP-C1	This work[a]
xDYRK1B (full length)	*Xenopus laevis*	NP_001080262.1	pCMV-SPORT6	Lilienthal et al. 2010
GFP-SF3B1-NT (1-492)	*Homo sapiens*	NP_036565.2	pEGFP-C1	De Graaf et al.
GST-rDYRK1A-ΔC (1-499)	*Rattus norvegicus*	NP_036923.1	pGEX-2TK	Himpel et al. 2000
GST-hDYRK1B-ΔC (1-454)	*Homo sapiens*	NP_004705.1	pGEX-2TK	Papenfuss et al. 2022
His_6-hDYRK1Acat (126–490)	*Homo sapiens*	NP_001387.2	pEXP17	Alexeeva et al. 2015
His_6-hDYRK1Bcat (78-451)[b]	*Homo sapiens*	NP_004705.1	pEXP17	Alexeeva et al. 2015
His_6-zDYRK1Bcat (153-517)	*Danio rerio*	XP_005158272.1	pEXP17	This work[a]

a, The cDNA sequence was subcloned from pCS2+DYRK1B (kind gift of Robert Nissen, Mazmanian et al. 2010). The protein encoded by this cDNA differs from the reference sequence by harboring a missense variant (K537N).

b, The protein expressed from this vector differs from the reference sequence by harboring a missense variant (L192M).

References for the cDNA clones:

Alexeeva M, Åberg E, Engh RA, Rothweiler U. The structure of a dual-specificity tyrosine phosphorylation-regulated kinase 1A-PKC412 complex reveals disulfide-bridge formation with the anomalous catalytic loop HRD(HCD) cysteine. *Acta Crystallogr D Biol Crystallogr* 71:1207-15 (2015).

Becker W, Weber Y, Wetzel K, Eirmbter K, Tejedor FJ, Joost HG. Sequence characteristics, subcellular localization, and substrate specificity of DYRK-related kinases, a novel family of dual specificity protein kinases. *J Biol Chem* 273:25893-902 (1998).

de Graaf K, Czajkowska H, Rottmann S, Packman LC, Lilischkis R, Lüscher B, Becker W. The protein kinase DYRK1A phosphorylates the splicing factor SF3b1/SAP155 at Thr434, a novel in vivo phosphorylation site. *BMC Biochem.* 7:7 (2006).

Himpel S, Tegge W, Frank R, Leder S, Joost HG, Becker W. Specificity determinants of substrate recognition by the protein kinase DYRK1A. *J Biol Chem.* 275:2431-8 (2000).

Leder S, Weber Y, Altafaj X, Estivill X, Joost HG, Becker W. Cloning and characterization of DYRK1B, a novel member of the DYRK family of protein kinases. *Biochem Biophys Res Commun* 254:474-9 (1999).

Lilienthal E, Kolanowski K, Becker W. Development of a sensitive non-radioactive protein kinase assay and its application for detecting DYRK activity in Xenopus laevis oocytes. *BMC Biochem* 11:20 (2010).

Mazmanian G, Kovshilovsky M, Yen D, Mohanty A, Mohanty S, Nee A, Nissen RM. The zebrafish dyrk1b gene is important for endoderm formation. *Genesis* 48:20-30 (2010).

Papenfuss M, Lützow S, Wilms G, Babendreyer A, Flaßhoff M, Kunick C, Becker W. Differential maturation and chaperone dependence of the paralogous protein kinases DYRK1A and DYRK1B. *Sci Rep.* 12:2393 (2022).

Table S2: Antibodies

Antibody target	Provider	Source/Clone	Dilution	Cat.-No.	RRID*
phospho-HIPK2 (Tyr361)	Thermo Fisher Scientific	Rabbit polyclonal	1:500	PA5-13045	RRID:AB_10987115
GST	GE Healthcare	Goat polyclonal	1:1000	27-4590	
GFP	Rockland	Goat polyclonal	1:1000	600-101-215	RRID:AB_218182
DYRK1B	custom made (Leder et al. 2003)[a]	Rabbit polyclonal	1:5000	n.a.	n.a.
Phospho SF3B1 (Thr434)	Custom made (de Graaf et al. 2006)	Rabbit polyclonal	1:200	n.a.	n.a.
HIF-1α	Cell Signaling Technology	Rabbit mAb D1S7W	1:1000	#36169S	RRID:AB_2799095
His6	GE healthcare	Mouse mAb	1:1000	27-4710	RRID:AB_771435
**Secondary HRP conjugated antibodies**					
Rabbit IgG(H+L)	Rockland	Donkey (Secondary)	1:2000	611-703-127	RRID:AB_218614
Mouse IgG	Invitrogen (Thermo Scientific)	Goat (Secondary)	1:2000	# 31430	RRID:AB_228307
Goat IgG(H+L)	Rockland	Donkey (secondary)	1:2000	605-703-125	RRID:AB_218291

Uncropped Western Blot Images

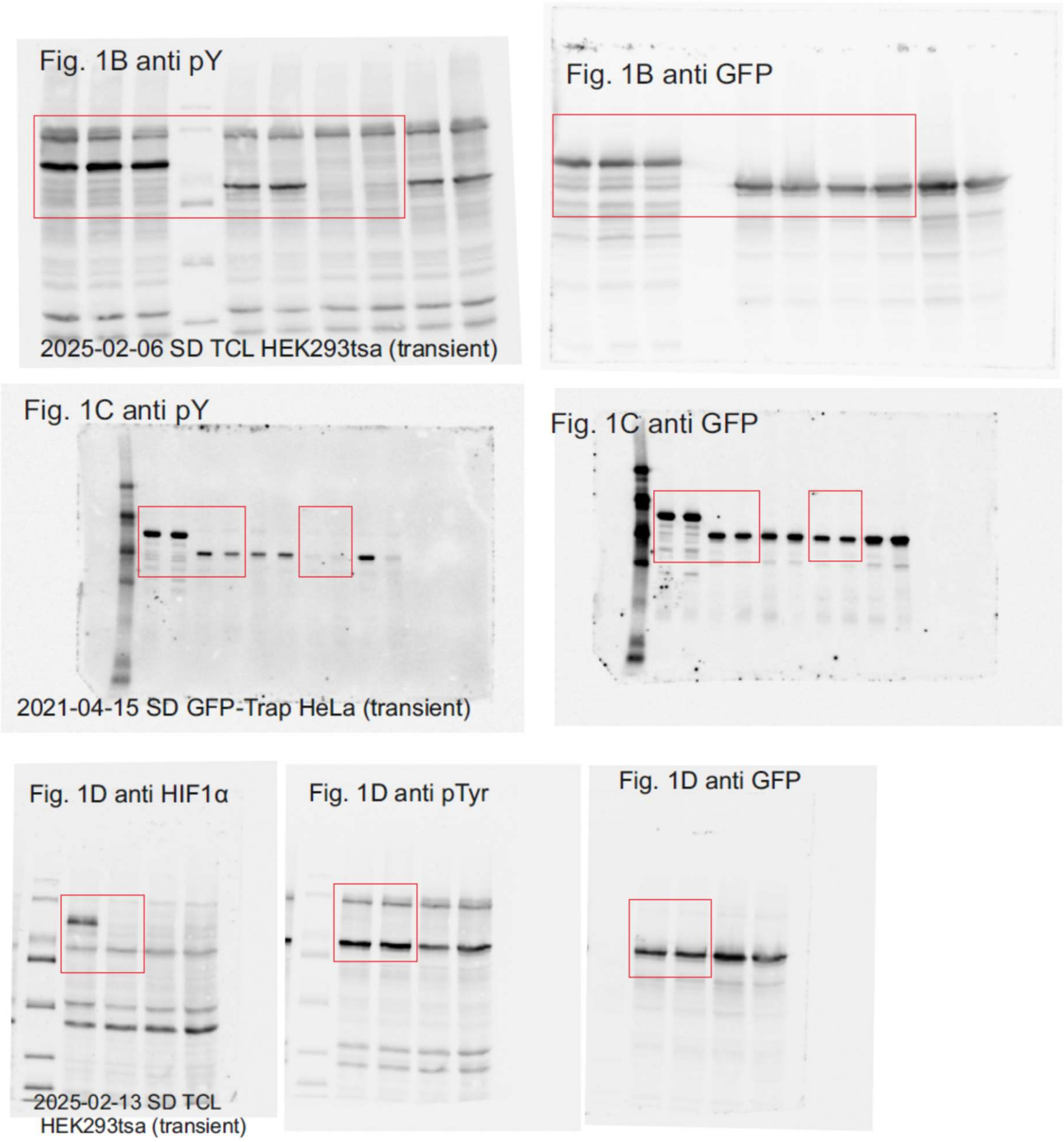

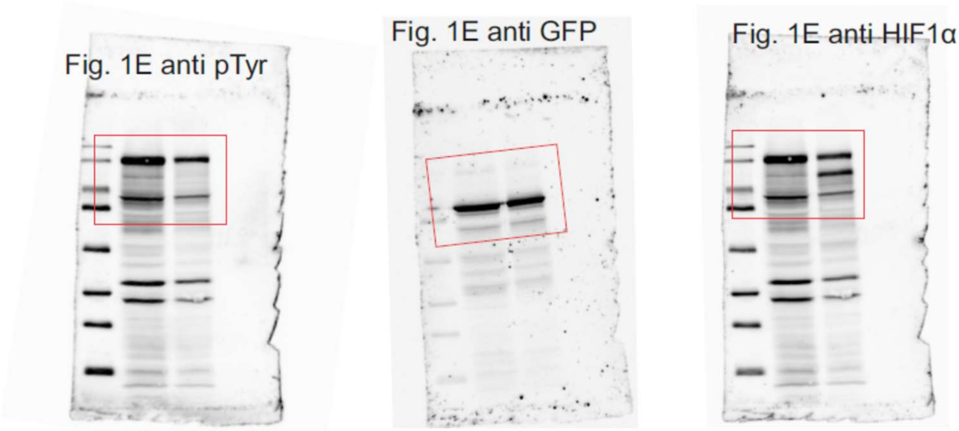

2025-06-24 PH TCL
HEK293tsa (transient)

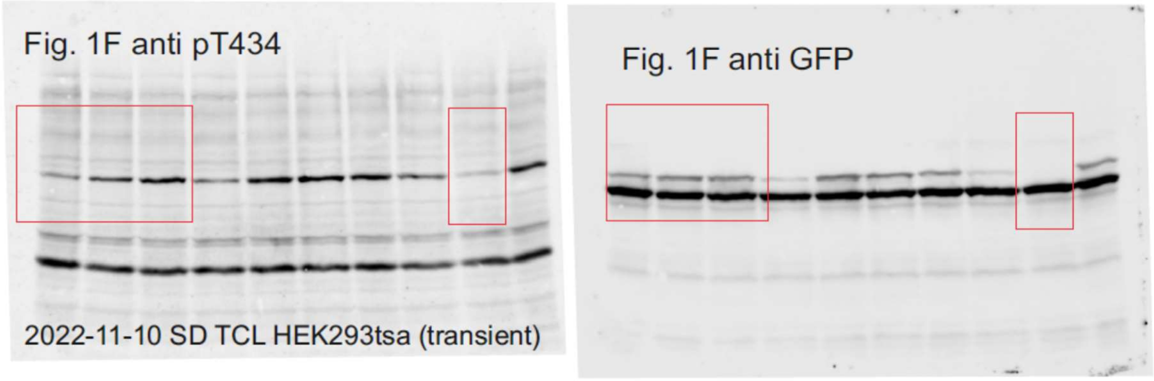

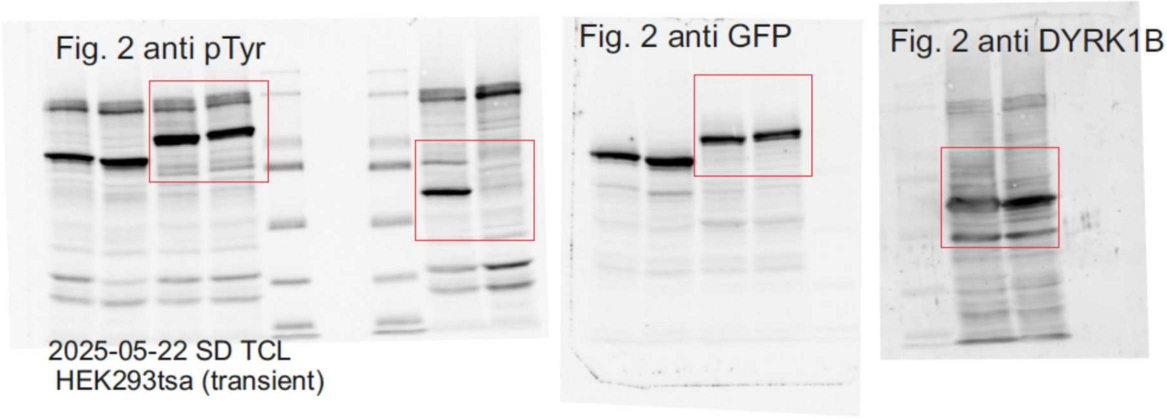

2025-05-22 SD TCL
HEK293tsa (transient)

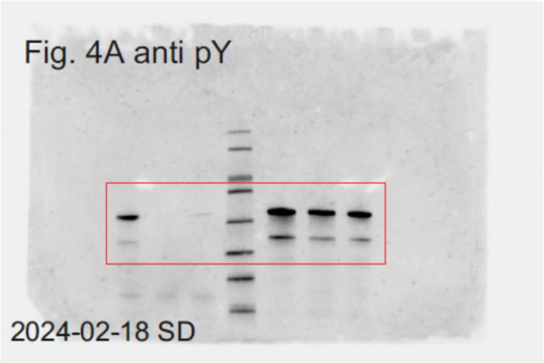
Fig. 4A anti pY
2024-02-18 SD

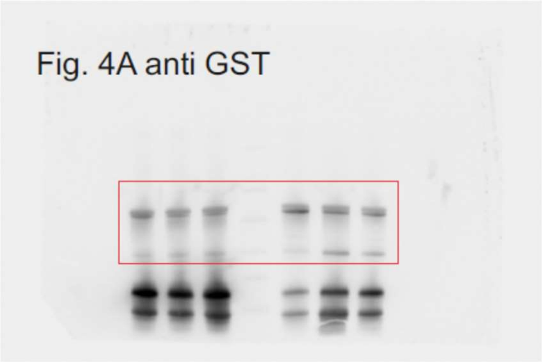
Fig. 4A anti GST

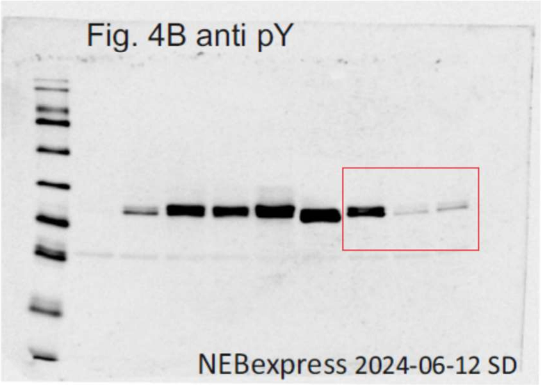
Fig. 4B anti pY
NEBexpress 2024-06-12 SD

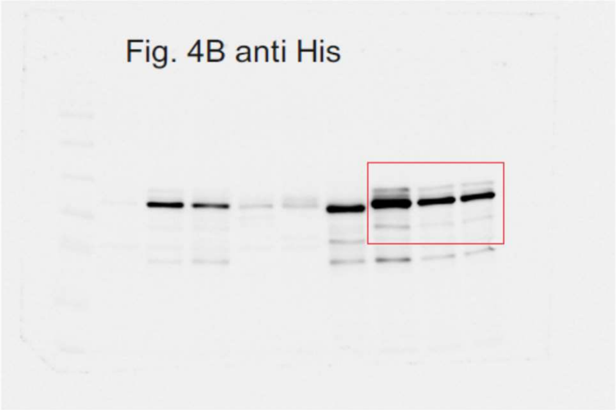
Fig. 4B anti His

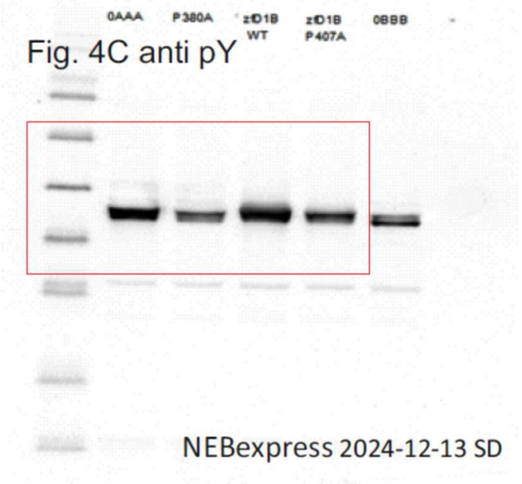
0AAA
P380A
zfD1B WT
zfD1B P407A
0BBB
Fig. 4C anti pY
NEBexpress 2024-12-13 SD

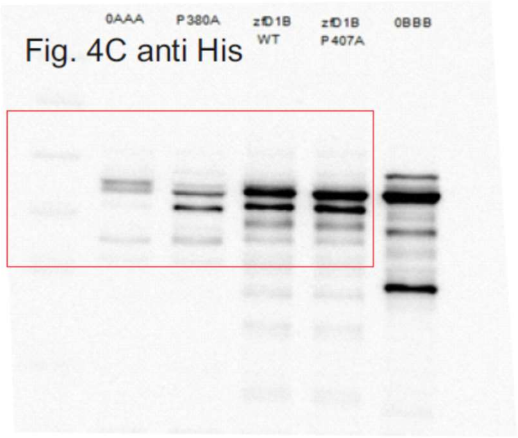
0AAA
P380A
zfD1B WT
zfD1B P407A
0BBB
Fig. 4C anti His

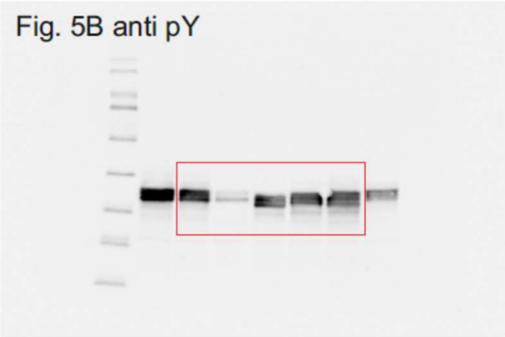

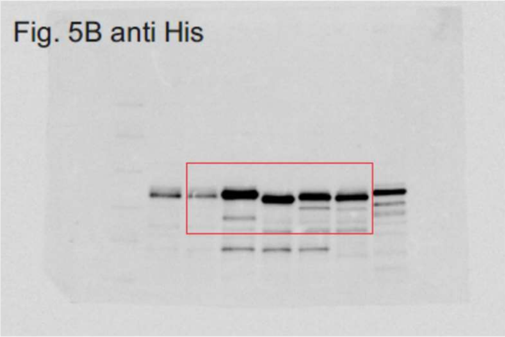

2023-09-28 NEBexpress SD

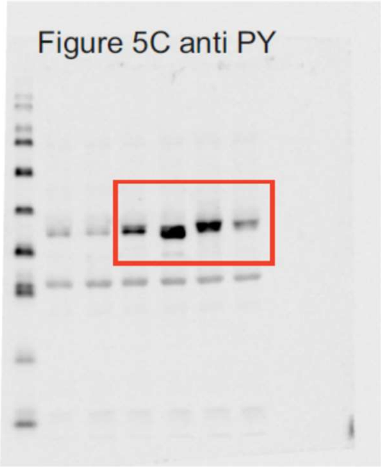

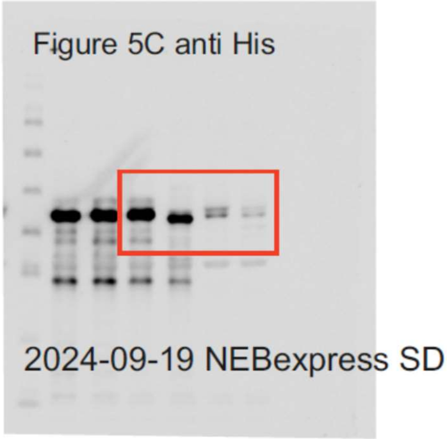

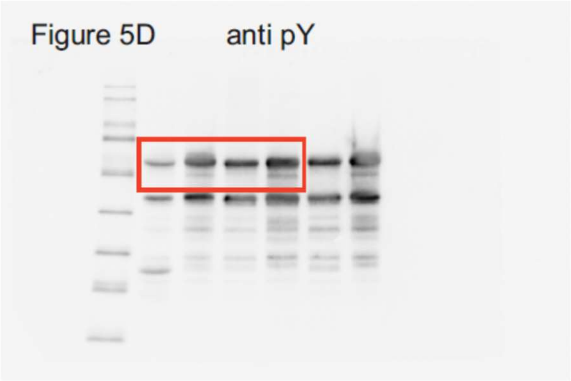

Figure 5D

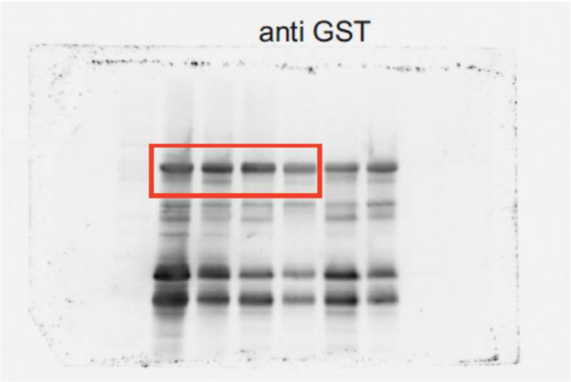

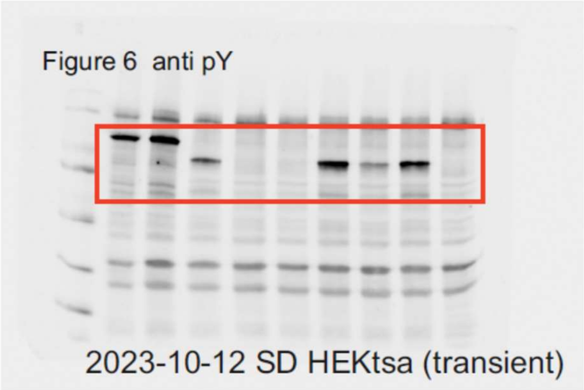
Figure 6 anti pY
2023-10-12 SD HEKtsa (transient)

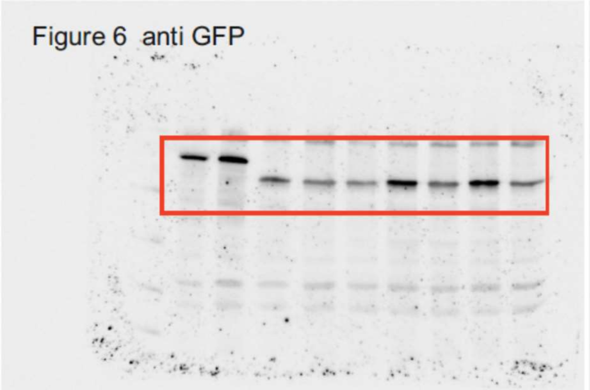
Figure 6 anti GFP

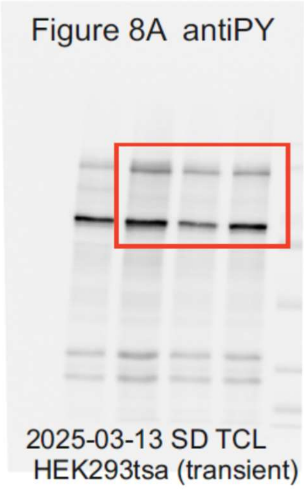
Figure 8A antiPY
2025-03-13 SD TCL
HEK293tsa (transient)

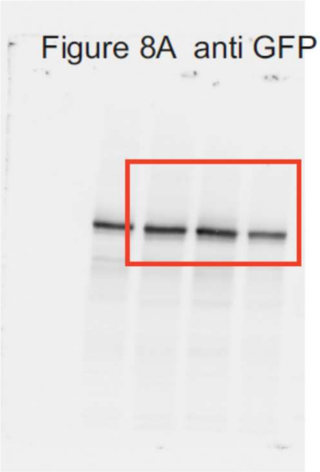
Figure 8A anti GFP

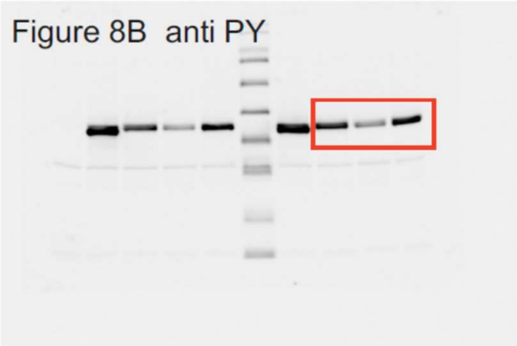
Figure 8B anti PY

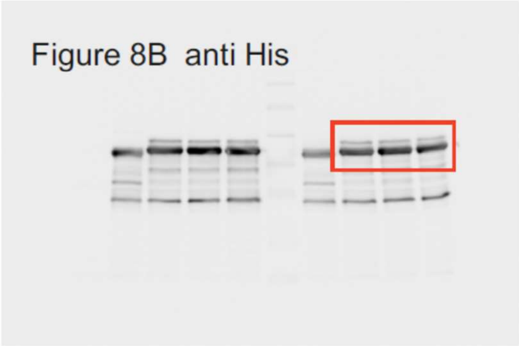
Figure 8B anti His

Figure S1A anti pY

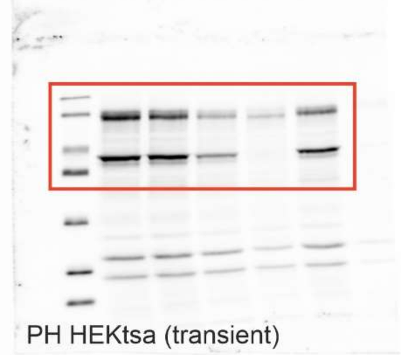

PH HEKtsa (transient)

Figure S1B anti pY

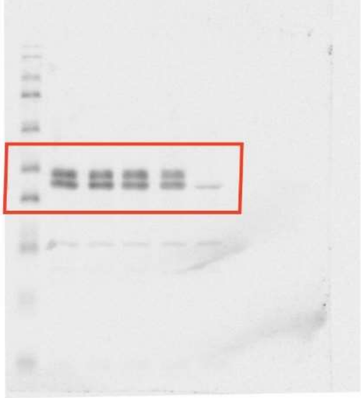

Figure S1B anti His

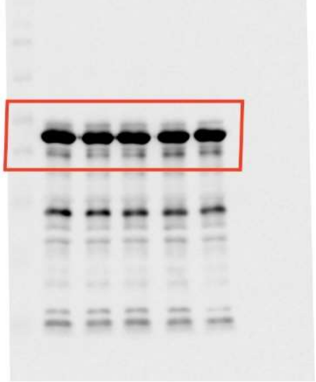

Figure S1A anti GFP

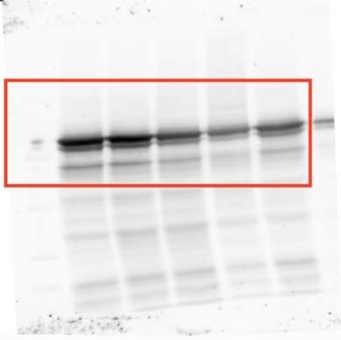

Fig. S2 anti pY

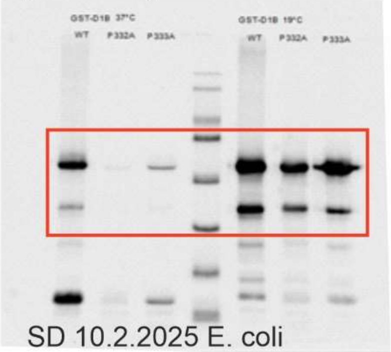

SD 10.2.2025 E. coli

Figure S1A anti HIF1

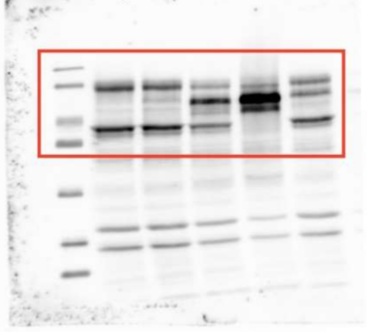

Fig. S2 anti GST

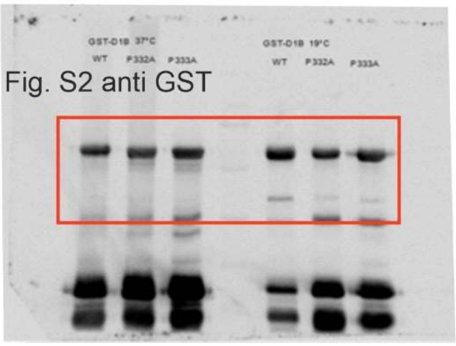

Supplement: Supplementary file 1 — Supplementary Material 1 [file 41598_2025_33562_MOESM1_ESM.pdf]
